# The CompTox Chemistry Dashboard: a community data resource for environmental chemistry

**DOI:** 10.1186/s13321-017-0247-6

**Published:** 2017-11-28

**Authors:** Antony J. Williams, Christopher M. Grulke, Jeff Edwards, Andrew D. McEachran, Kamel Mansouri, Nancy C. Baker, Grace Patlewicz, Imran Shah, John F. Wambaugh, Richard S. Judson, Ann M. Richard

**Affiliations:** 1 National Center for Computational Toxicology, Office of Research and Development, U.S. Environmental Protection Agency, Research Triangle Park, NC USA; 2 Oak Ridge Institute for Science and Education, Oak Ridge, TN USA; 3 Research Triangle Park, NC USA; 4 ScitoVation LLC, Research Triangle Park, NC USA

**Keywords:** Environmental chemistry, Computational toxicology, Compound database, Data curation, Open data, Physicochemical properties, Environmental fate and transport data, Bioassay data, Toxicity data, Non-targeted analysis, Toxic Substances Control Act (TSCA), ToxCast, ToxRefDB, EDSP21

## Abstract

Despite an abundance of online databases providing access to chemical data, there is increasing demand for *high*-*quality, structure*-*curated, open data* to meet the various needs of the environmental sciences and computational toxicology communities. The U.S. Environmental Protection Agency’s (EPA) web-based CompTox Chemistry Dashboard is addressing these needs by integrating diverse types of relevant domain data through a cheminformatics layer, built upon a database of curated substances linked to chemical structures. These data include physicochemical, environmental fate and transport, exposure, usage, in vivo toxicity, and in vitro bioassay data, surfaced through an integration hub with link-outs to additional EPA data and public domain online resources. Batch searching allows for direct chemical identifier (ID) mapping and downloading of multiple data streams in several different formats. This facilitates fast access to available structure, property, toxicity, and bioassay data for collections of chemicals (hundreds to thousands at a time). Advanced search capabilities are available to support, for example, non-targeted analysis and identification of chemicals using mass spectrometry. The contents of the chemistry database, presently containing ~ 760,000 substances, are available as public domain data for download. The chemistry content underpinning the Dashboard has been aggregated over the past 15 years by both manual and auto-curation techniques within EPA’s DSSTox project. DSSTox chemical content is subject to strict quality controls to enforce consistency among chemical substance-structure identifiers, as well as list curation review to ensure accurate linkages of DSSTox substances to chemical lists and associated data. The Dashboard, publicly launched in April 2016, has expanded considerably in content and user traffic over the past year. It is continuously evolving with the growth of DSSTox into high-interest or data-rich domains of interest to EPA, such as chemicals on the Toxic Substances Control Act listing, while providing the user community with a flexible and dynamic web-based platform for integration, processing, visualization and delivery of data and resources. The Dashboard provides support for a broad array of research and regulatory programs across the worldwide community of toxicologists and environmental scientists.

**Electronic supplementary material:**

The online version of this article (10.1186/s13321-017-0247-6) contains supplementary material, which is available to authorized users.

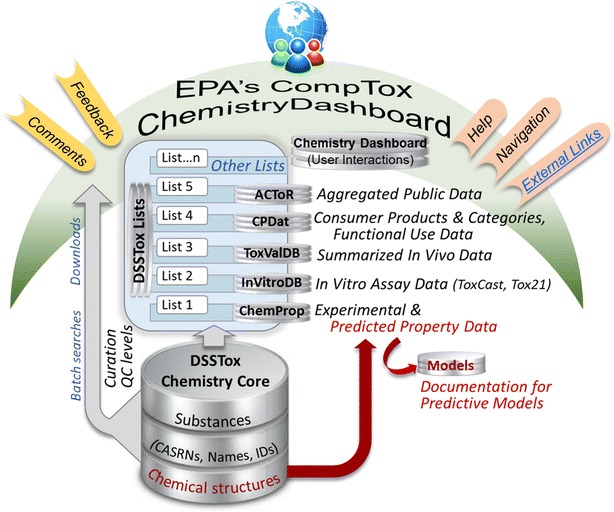

## Background

There are currently many open sources of chemistry and biology data serving a broad range of scientific disciplines and needs [1]. While early internet chemistry databases delivered limited data for hundreds to thousands of chemical structures, advances in modern Internet technologies had enabled an explosion of freely available online chemistry data over the past decade. PubChem [2] currently provides chemical content for ~ 94 million compounds, ChemSpider [3] serves up data for 59 million structures, and dozens of other chemistry databases serve up smaller, but often more focused datasets that have high visibility in the community. Examples of these smaller data sets include ChEMBL [4, 5] and the Human Metabolome Database [6, 7]. An exhaustive listing of available databases is outside the scope of this article, but interested readers are referred to Wikipedia Chemistry Databases [8] as a good *starting point* to research the range of chemical databases available online.

Several resources already exist in the domains of computational toxicology and environmental science, some of which provide rich data streams, predictive models and online tools of use to these communities. For example, the Organization for Economic Cooperation and Development (OECD) has developed eChemPortal [9], which provides free public access to chemicals and associated properties, allowing searches by chemical name and number, by chemical property, and by Global Hazard Summary (GHS) classification. The site provides access to collections of chemical hazard and risk information that have been prepared for government chemical review programs worldwide. Of particular note is the European Union’s (EU) Chemicals Association’s ECHA CHEM database [10], which is comprised of information submitted for chemical substances registered under the registration, evaluation, authorization and restriction of chemicals (REACH) regulation [11] as well as information in the ECHA C&L (Classification and Labeling) Inventory [12]. The quantitative structure–activity relationship (QSAR) application, known as the OECD QSAR Toolbox [13], directly interacts with the eChemPortal database providing many cheminformatics functions that facilitate data access and usage.

Similarly, the U.S. Environmental Protection Agency (EPA) strives to make its data and models publicly available to support the regulatory and scientific communities’ efforts to evaluate chemicals [14]. Several of these resources are focused on human health risk assessment [15] and “safer” chemicals [16]. Modelling tools have been specifically developed to support the EPA’s Toxic Substances Control Act (TSCA) program such as the Estimation Prediction Interface (EPI) from the EPI Suite tool [17]. Researchers within EPA’s National Center for Computational Toxicology (NCCT) have developed several databases and web-user interfaces (i.e., dashboards) over the years with similar intent, including the ToxCast Dashboard [18], the Endocrine Disruption Screening Program (EDSP) for the 21st Century (EDSP21) Dashboard [19], the Chemical and Product Categories database (CPCat) [20], and the Aggregated Computational Toxicology Online Resource (ACToR) [21]. Collectively, these applications have delivered access to in vitro bioassay data [22], chemical and product categories information [23, 24], exposure data [25, 26], experimental and predicted physicochemical property data [27, 28] and, with ACToR [29], thousands of toxicity testing results aggregated from more than 1000 public sources for over 500,000 chemicals.

A major goal of EPA’s Chemical Safety for Sustainability research program has been to develop capabilities that allow rapid and cost-effective evaluation of large numbers of chemicals for potential adverse effects and risk to humans and ecosystems. NCCT’s ToxCast program [30], and the affiliated multi-agency Tox21 program [31], are engaged in the generation and analysis of in vitro bioassay data for thousands of chemicals evaluated in hundreds of high-throughput and high-content screening (HTS and HCS) assays. Beyond hazard evaluation and prioritization, innovative methods for rapid exposure and dose assessments are also being developed [23–26]. NCCT research includes the development of various models for predicting physicochemical properties [27, 28], activity at various enzyme targets and for cell-based outcomes, pharmacokinetics parameters [32], and exposure [33, 34]. These research efforts are combined using chemistry and the DSSTox database as an integration platform that brings together the data associated with the various research efforts into a single web-based application.

The original Distributed Structure-Searchable Toxicity (DSSTox) web application, launched in 2004, provided a common access point for several thousand environmental chemicals associated initially with four publicly available toxicity datasets pertaining to carcinogenicity, acute aquatic fish toxicity, water disinfection by-products, and estrogen-receptor binding activity [35, 36]. These DSSTox data files provided, for the first time, highly-curated and standardized chemical structures linked to bioactivity data that served as an essential resource for structure–activity relationship (SAR) model development. The quality of mappings between chemical substance identifiers (e.g., Chemical Abstracts Service Registry Numbers, or CASRN, and names) and their corresponding structures yielded a unified DSSTox structure index for chemical-data sources. DSSTox continued to expand over the next decade with web publication of additional chemical structure files for sets of interest to the toxicology and environmental science communities (see [37] for more information).

From 2007 onward, the DSSTox database was enlisted to serve as the cheminformatics backbone of the NCCT’s ToxCast and the multi-agency Tox21 HTS screening programs, with DSSTox curators registering more than 8000 unique chemical substances corresponding to test samples entering one or both screening libraries. Richard et al. [38], provides an account of the evolution and application of the chemical library for the ToxCast program]. By mid-2014, the manually curated DSSTox database had grown to approximately 25,000 chemical substances, spanning more than a dozen inventories. Despite this growth, DSSTox provided only partial coverage of larger, chemical inventories (e.g., the more than 80,000 substances in the TSCA inventory [39], and tens of thousands of substances in the EDSP universe [40]).

The focused nature of DSSTox was in part dictated by the constraints of the manual curation efforts, which ensured high quality structure-identifier mappings. However, this approach was too resource intensive for expansion to the very large chemical inventories important to regulatory authorities in the US and worldwide (for example for EChA [41] and Health Canada [42]). Whereas a number of large chemically-indexed databases (such as PubChem, ChemSpider, ChEMBL, ChemIDPlus, and ACToR) were providing sources of additional chemical structures and identifiers, DSSTox’s historical curation efforts encountered high rates of inaccuracies and mis-mapped chemical identifiers in these public domain sources (e.g., a name or registry number incorrectly mapped to one or more structures). This is a well-recognized problem that has been documented in some detail by others [43, 44]. As such, a strategy was developed to expand DSSTox by adding data available from publicly available data sources, while also controlling for the limitations of those sources and preserving the aspects of quality curation upon which DSSTox was built.

The product of this database expansion effort was developed using both manual and algorithmic curation techniques. A key constraint applied to this expansion of DSSTox was the requirement for a 1:1:1 mapping among the DSSTox preferred name for a chemical (chosen to be unique), the active (or current) CASRN, and the chemical structure, as could be uniquely rendered in a mol file format. Subject to these constraints (i.e., disallowing conflicts) chemical structures and uniquely mapped identifiers were sequentially loaded into DSSTox from the following public databases:

1. the EPA Substance Registry Services (SRS) database (containing the public TSCA chemical inventory, accessed at [45]);
2. the National Library of Medicine’s (NLM) ChemIDPlus (part of the TOXNET suite of databases, accessed at [46]);
3. a portion of the National Center for Biotechnology Information’s (NCBI) PubChem database, i.e., the approximately 700,000 subset containing registry number identifiers, along with other chemical identifiers, accessed at [2]).

Based on the number of sources that agreed on mappings of identifiers to structures, these public data were loaded with a defined quality control annotation (qc_level) [38]. There are 2 DSSTox (manual curation) levels and 3 Public (auto-curation) levels ranging from low to high as defined in Table 1. A fourth auto-curation level not included in the table, termed as “incomplete” in the DSSTox database, includes partially mapped chemicals programmatically extracted from public domain databases but deemed to not be of sufficiently high-quality to release to the public.

**Table 1:** The description of different curation qc_levels

Level	Description
1	Expert curated: highest confidence in accuracy and consistency of unique chemical identifiers
2	Expert curated: unique chemical identifiers confirmed using multiple public sources
3	Programmatically curated from high quality EPA source(s) and unique chemical identifiers have no conflicts in ChemIDPlus and PubChem
4	Programmatically curated from ChemIDPlus. Unique chemical identifiers have no conflicts in PubChem
5	Programmatically curated from ACToR or PubChem. Unique chemical identifiers have low confidence and have a single public source

In addition to the programmatic incorporation of non-conflicting portions of SRS, ChemIDPlus and PubChem into DSSTox, both manual and programmatically assisted curation has continued to address critical gaps in coverage of high-interest environmental lists, including pesticides, food additives, chemicals of potential concern for endocrine disruption, chemicals with known functional use in products, and substances on the public EPA hydraulic fracturing chemicals list [47]. With these latest additions, the DSSTox database currently exceeds 760,000 substance records, with more than 60,000 manually curated (the sum of Level 1 and 2 data quality), or having consistent identifier assignments in three or more public databases (Level 3), constituting the highest qc_levels content. The clean mapping of structural identifiers (names, CASRN) to chemical structures, with a quality control annotation, provides an essential underpinning to cheminformatics workflows in the Dashboard.

Driving principles across all of EPA’s research programs are the application of quality metrics and data standards, and the use and dissemination of open, public data and tools to maximize the scientific reach, utility, and outcomes of our work. These data and capabilities, in turn, support the EPA mission to develop prediction models and methods to inform various regulatory needs, from priority setting, to screening level hazard assessment, to chemical risk assessment. These representative research efforts, and many others through collaborations within and outside of EPA, depend on the aggregation of various data types into structured databases that can be queried and integrated in meaningful ways. This requires an informatics foundation providing for the storage of chemical structures, the accurate mapping of structures to data, and the integration of the various types of data of interest to both our research and to that of the wider scientific community [38].

This work reports on the EPA’s CompTox Chemistry Dashboard (hereafter referred to as the “Dashboard”), a publicly available, web-based application that provides a portal into the EPA’s growing inventory of chemical databases and capabilities, providing access to data of interest to environmental chemists and toxicologists, and tools to support computational toxicology research. These data are generated within the EPA, as well as harvested and aggregated from public domain resources and collaborations. The Dashboard is the latest public web interface developed by EPA for the dissemination of our data to the public, superseding and providing web-portal entry into legacy databases and tools, all of which now “sit atop” a uniform chemical database infrastructure. The Dashboard additionally provides an internal, standardized, multipurpose EPA development platform for adding new databases and functional modules, many of which are currently available for internal use by EPA researchers and program office representatives and, once reviewed and tested, are then released to the community through the Dashboard. Elements of such workflows, designed to support predictive modeling approaches as part of EPA’s ToxCast and ExpoCast programs, are currently being surfaced through the Dashboard.

## Methods and results

The Dashboard is a freely accessible web-based application and data hub providing access to data associated with almost 760,000 chemical substances. It accesses data from nine component databases housing generic data types (listed in Table 2). The Dashboard also integrates data from other platforms (specifically PubChem and PubMed, as discussed in more detail later) via web services and visualization widgets. The Dashboard represents a first step in building a comprehensive chemical-substance-centric informatics architecture to provide flexible access to data, models and analysis tools in support of EPA’s research programs.

**Table 2:** The list of all databases underlying the CompTox Chemistry Dashboard, identified by the database name and data types contained within each database

Database name	Data type
DSSTox-Core	Chemical structures, identifiers
DSSTox-Lists	Chemical mappings
DSSTox-ChemProp	Experimental and predicted property data
DSSTox-Models	Documentation for predictive models
InVitroDB	In vitro assay data
ACToR	Aggregated public data
ToxValDB	Summarized in vivo data
CPDat	Consumer products and categories data, functional use data
ChemDashboard	Comments, feedback, help and navigation, external links

### DSSTox database assembly (comprised of Core, Lists, ChemProp and Models databases)

Consistent with the Dashboard being a chemical-centric application, the DSSTox database assembly is the *primary* set of databases underpinning the Dashboard. Chemical substances surfaced via the Dashboard are stored in the DSSTox database with associated identifiers (e.g., CASRN, systematic and trivial names).

Historically, DSSTox evolved with a focus on curating chemical information associated with public datasets of high interest to the environmental toxicology community [48]. To enable that curation effort, DSSTox has three primary entities, each labeled with a permanent intransient DSSTox Identifier. ***DSSTox*** **-** ***Core*** consists primarily of the first two of these identifiers: ***DTXSIDs*** are unique substance identifiers, where a substance can be any single chemical, mixture, polymer (e.g., Polyvinyl chloride, [49]) or chemical family (e.g., Polychlorinated biphenyls [50]) and ***DTXCIDs*** are unique (as determined using InChI Keys) identifiers of chemical structures. DSSTox-Core provides what is considered “truth” regarding a chemical substance; curators manually verified the consistent and appropriate mapping of names, registry numbers and structure (a DTXCID) for the core substance records (DTXSIDs), while denoting the *qc_level* confidence in the mappings. In addition, linkages between chemicals are manually annotated to provide context or “representative” examples when a structure cannot be drawn (vide infra), a registry number cannot be found in public data, or a registry number may not exist. All structures in the database are managed primarily using cheminformatics functions from ChemAxon’s [51] JChem Java API [52] for structural conversion, image generation, mass and formula calculations. The Indigo Toolkit v1.2.1 [53] is employed to generate standard InChIs and InChI keys. ACD/Labs Name Batch v2016.2.2 [54] is used to generate IUPAC and Index Names (based on IUPAC and Chemical Abstracts Service nomenclature rules) for our chemical structures.

In addition to the substance and structure identifiers in DSSTox-Core, a third identifier, the DTXRID resides in the DSSTox-List database. DTXRIDs are unique identifiers of source substances, mapping to a substance record from an external source list carrying all source-associated identifiers. When a new external source of data is loaded into DSSTox, the linkage between a particular DTXRID and an associated DTXSID is established using a programmatic mapping script that compares each identifier attached to the DTXRID to the chemical identifiers attached to the DTXSID. Potential hits are scored based on the type of identifier match, and for name based matches, the confidence in the mapping between the name and DTXSID during core data curation is noted. The highest scoring DTXSID is tentatively linked, and in cases where the data is of high interest, curators review the mappings to verify or correct.

**Fig. 1:**
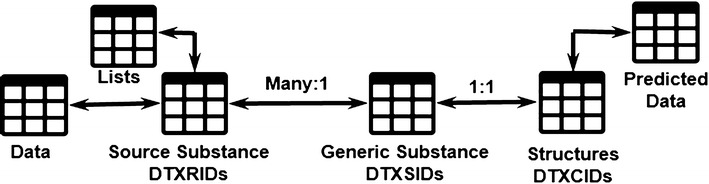
The relationship between the various identifiers in the DSSTox Database

The DSSTox Chemical Property database (DSSTox-ChemProp) was built as an add-on to DSSTox-Lists to capture measured or predicted property data associated with a particular source substance or list of chemicals (DTXRIDs). In many cases, public data are collected from external sources to be subsequently utilized for QSAR model building. These QSAR models, once built, are used to predict values for the structural content (DTXCIDs). Since these predicted values are closely tied to the public data already being stored in DSSTox-ChemProp, predictions are also stored in the ChemProp data tables. To ensure that all associated information for the models and their predictions are captured (e.g., descriptor values, statistics, methods and versions of the models used to make those predictions), the predicted values in ChemProp have been supplemented by the development of the DSSTox-Models database. The relationship between the different identifiers is illustrated diagrammatically in Fig. 1.

The CPDat, ToxValDB, ACToR, InVitroDB databases shown in individual tabs in the Dashboard, and the original DSSTox database (vide supra) were designed and developed by EPA researchers to address varied agency needs. Although the development of these individual resources was originally carried out with varying degrees of coordination to the DSSTox project, all are either fully mapped (InVitroDB) or content was auto-mapped to the DSSTox database to the extent possible while disallowing identifier conflicts (see above). The result has been incomplete DSSTox mapping coverage in the case of ACToR and CPDat, where further curation efforts to resolve conflicts in public domain chemical IDs will be addressed in the future.

### InVitroDB

The ToxCast and Tox21 in vitro high-throughput screening (HTS) programs have generated data for ~ 10,000 compounds in hundreds of assays [55]. Note that not all chemicals are measured in all assays. InVitroDB is an EPA database built to store and facilitate the analysis of that screening data, with the raw HTS data processed through the ToxCast pipeline (*tcpl*) [56]. With a multilevel approach to clearly demarcate the changes caused by different transformations, the database provides data from raw assay readouts through controled normalization and fitting of the concentration response with three methods to final hit calls and quality flags conveying curve fit concerns. Static versions of this internal database [57] are released to the public at regular intervals.

### ACToR

ACToR contains knowledge extracted from large collections of data and data sets that are transformed into computable formats [58]. ACToR’s simple and flexible data model enables it to store nearly any data associated with a chemical. The current release of ACToR contains nearly 560,000 chemical entities (as defined by CASRN) and content from roughly 2700 data collections. Each of these data collections is extracted and translated from the original source to a common data format by expert curators following well-defined procedures. Prior to the 2014 expansion of DSSTox, ACToR’s CASRN-chemical content far exceeded that of DSSTox, with the non-overlapping portion not subject to DSSTox’s strict chemical structure-curation standards. With the expansion of DSSTox, a much larger proportion of ACToR (> 50%) has been cleanly mapped to DSSTox chemical content (i.e., with no conflicts) and this mapped portion of ACToR has fed content to many of EPA’s other databases: the physicochemical property data were moved to DSSTox-ChemProp, in vitro assay results were transferred to InVitroDB, and in vivo toxicity data were loaded into ToxValDB (vide infra). However, a significant volume of data remains in ACToR alone due to the heterogeneity of the data collected. Chemical and data curation efforts are ongoing to prioritize and more fully incorporate ACToR chemical-data content into EPA’s databases.

### ToxValDB

The need for organized in vivo toxicity data to evaluate alternative in vitro and in silico approaches led to the development of the ToxRefDB database to house a detailed collection of animal toxicity study data, primarily extracted from EPA pesticide registration documents [59]. The database is highly-structured, consisting of data extracted from thousands of studies on over 1000 chemicals, thus comprising one of the largest in vivo toxicity databases available to the public. The restrictions on transparency, study rigor, and required detail in ToxRefDB maintain a very clean and valuable database, but prevent the integration of less detailed data from many other sources. ToxValDB is a database designed to store a wider range of public toxicity information in a less restricted, more summarized form than ToxRef, while maintaining the linkages to original source information so that users can access available details.

In particular, ToxValDB collates publicly available toxicity dose–effect related summary values typically used in risk assessments. These include Point of Departure (POD) data collected from data sources within ACToR and ToxRefDB, and no-observed and lowest-observed (adverse) effect levels (NOEL, NOAEL, LOEL, LOAEL) data extracted from repeated dose toxicity studies submitted under REACH. Also included are reference dose and concentration values (RfDs and RfCs) from EPA’s Integrated Risk Information System (IRIS) [60] and dose descriptors from EPA’s Provisional Peer-Reviewed Toxicity Values (PPRTV) documents [61]. Acute toxicity information was extracted from a number of different sources, including: OECD eChemPortal, ECHA (European Chemicals Agency), NLM (National Library of Medicine) HSDB (Hazardous Substances Data Bank), ChemIDplus via EPA TEST (Toxicity Estimation Software Tool), and the EU JRC (Joint Research Centre) AcutoxBase [62]. Finally, data from the eChemPortal and the EU COSMOS project have also been included in ToxValDB.

### CPDat

EPA researchers have aggregated data on consumer product composition in a number of databases: the Chemical/Product Categories database (CPCat) [20], the Consumer Product Chemical Profiles database CPCPdb [24], and the functional use of chemicals database (FUse DB) [63, 64]). These data have now been fully consolidated into the Chemicals and Products Database (CPDat) [65, 66] using a consistent scheme for categorizing products and chemicals. CPDat also includes a number of newly acquired data sources on product composition (both reported values and quantitative predictions based on ingredient list labels) and functional use. The current version of CPDat contains reports on over 75,000 chemicals that are listed as constituents in one or more of 15,000 consumer products [23]. Although the data’s primary intended use is to inform exposure, risk, and safety assessments, it also has served as a resource for building computational models to predict weight fractions and functional use of chemicals based on structure [63]. These models, in turn, have been used to more broadly populate these vital data for a much broader set of chemicals (~ 30,000 DSSTox structures currently) to inform exposure and risk assessors evaluating chemical and product safety.

### ChemDashboard

The ChemDashboard database is an internal-to-EPA application support database providing the necessary infrastructure to support the function of the Dashboard application rather than the data displayed in the application. The Dashboard has built-in administrative functions that allow an administrator to add hypermedia links to external information, control the display of tabs and data, and manage the list content available in the interface in the production version of the dashboard. All of the options and parameters to control the interface are stored in the ChemDashboard database. In addition, help and informational text can be edited through the administrative panel and fed directly into the database. Since these data are not coded into the application but modified through the administrator panel this means that they can be added between new releases of the application. The ChemDashboard database is also the container for comments [67] and feedback from the user community, including both application improvement ideas for the development team and crowdsourced curation recommendations for the other integrated databases.

## Application implementation

The Dashboard project began in late 2015 and, to facilitate rapid development of a production application, has been developed as a “Ruby on Rails” application built on top of a set of MySQL and PostgreSQL databases, using Agile development practices. The application is therefore principally a 2-tiered architecture.

The current version of the Dashboard is using Ruby 2.4.1 [68] and Ruby On Rails 4.2.8 [69]. HTML5, Cascading Style Sheets (CSS) and javascript libraries were used to construct the user interface and generate a cohesive user experience. Portions of the interface are supported via RESTful web service endpoints provided as part of the ACToR web services project [70]. Cheminformatics functions in the Dashboard application are carried out using the Indigo toolkit and similarity searching is enabled via the epam Bingo PostGreSQL cartridge [71].

## The CompTox Chemistry Dashboard web-based application

**Fig. 2:**
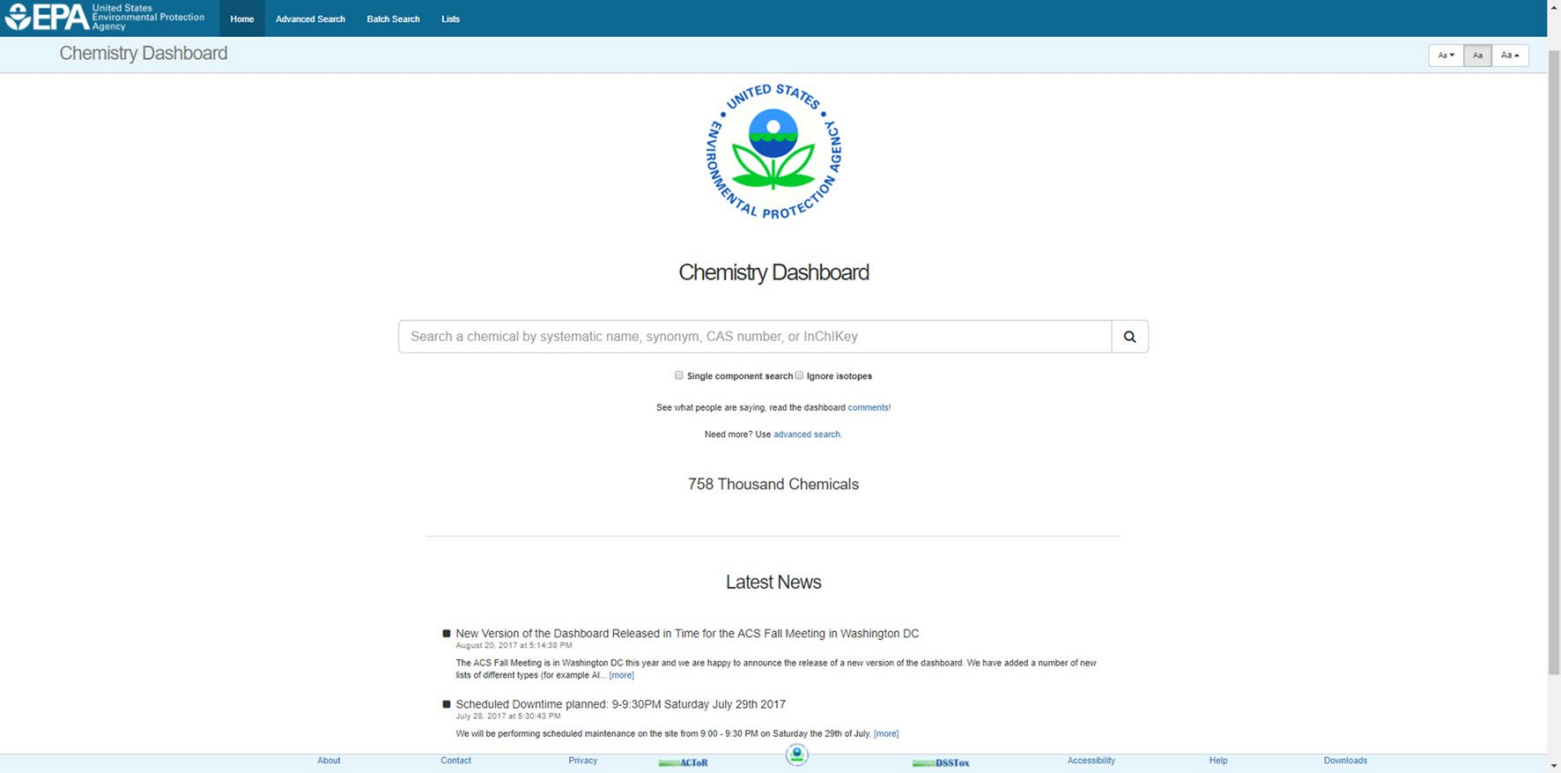
The CompTox Chemistry Dashboard entry page. Searches can be performed based on chemical names, CASRNs and InChIKeys, with pre-filters to select single component chemicals and to ignore chemicals with isotopes. The home page also provides “Latest News” updates

The initial landing page for the Dashboard is a search box allowing a single chemical search using a simple alphanumeric text entry box (Fig. 2).

**Fig. 3:**
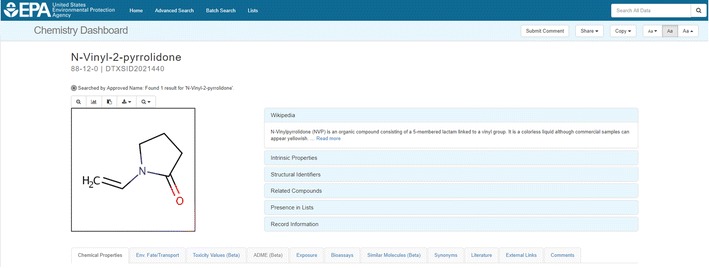
The header for a chemical details page. Details regarding the individual accordions (in blue, to the right of the structure) are described in the text. The tabs below the chemical header are greyed out when data are not available, in this case, ADME (adsorption, distribution, metabolism, elimination) data were unavailable

A successful search results in a chemical page header (Fig. 3) that displays:

1. a chemical structure (downloadable as a molfile or image);
2. intrinsic properties (e.g., molecular formula and monoisotopic mass);
3. chemical identifiers (e.g., systematic name, SMILES string, InChI string, and InChIKey);
4. related compounds (based on molecular skeleton search, molecular similarity search, and the presence of the chemical in mixtures or as salt forms);
5. one or more lists in which the chemical is present (e.g., ToxCast and Tox21); and
6. a record citation including a unique DSSTox substance identifier (DTXSID).

Below the header are a series of individual data tabs for a particular chemical. Tabs that are differentiated by blue fonts are active and indicate that data are available. Tabs that are greyed-out indicate no data are available. For chemicals where there is an abundance of publicly available data (for example, Atrazine [72]), all tabs are active and contain data. However, other chemicals, such as Domoic Acid [73], only return predicted chemical and environmental fate and transport property information, synonyms, external links, literature, and comments as active tabs. A description of the possible contents of each of these data tabs for a chemical search result will be discussed separately below.

### Chemical properties

**Fig. 4:**
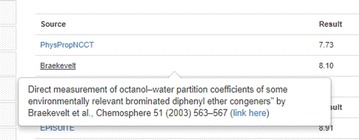
Experimental logP data included in the dashboard is linked to the original source publication using a DOI link [76]

The Chemical Properties tab contains experimental and predicted physicochemical properties sourced from a number of different online databases or predicted using different models detailed below. Physicochemical properties listed include log octanol–water partition coefficient (logP), water solubility (S), melting point (MP), and more than a dozen additional endpoints. The data are listed in two separate tables, divided into Experimental and Predicted data. The bulk of the available experimental data resulted from previous work curating the publicly available PHYSPROP datasets [74] using a combination of manual and automated workflows [27]. The largest set is for logP, which contains data for 14,050 chemicals, while the smallest set for Biodegradation Half-Life contains 150 chemicals. These curated data were used to develop the OPEn structure–activity Relationship Application (OPERA) [28] models, as well as to provide data for development of six NICEATM (NTP Interagency Center for the Evaluation of Alternative Toxicological Methods) models (vide infra). Based on feedback from the user community regarding failure of the initially published model results for particular classes of chemicals and endpoints of interest, additional data were extracted from the literature and added to the experimental property database. For example, user feedback indicated that the OPERA logP predictions for polybrominated diphenyl ether (PBDE) flame retardants were significantly underestimated. The addition of logP data for 9 PBDE congeners [75] and retraining of the models resulted in more accurate predictions for these 9 PBDEs and, not surprisingly, for the remaining 200 congeners as well. When data such as these are added to the training set, the Dashboard experimental data are updated with DOIs linking to the source publication (see Fig. 4).

Predicted data in the Dashboard have been generated using TEST, OPERA and ACD/Labs prediction models, or have been harvested from public websites in accordance with their data policies. Each source is detailed below.

1. *OPEn structure*–*activity Relationship Application Models (OPERA)* The OPERA models were developed using curated versions of the PHYSPROP datasets and k-nearest neighbor (kNN) QSAR modeling approaches. The models were developed based on the OECD principles for QSARs [77], with the intention of providing full transparency to users of the Dashboard, including generation of a report adhering to OECD QSAR Model Reporting Format (QMRF) guidelines [78]. Details of the model development are provided along with a Calculation Report for each chemical prediction; the latter reporting model performance statistics within both local and global applicability domains, as well as metrics for determining confidence in the chemical prediction. Up to 5 nearest-neighbors are displayed in the interface together with their experimental and predicted results for comparison. Figure 5 illustrates the Calculation Report for the logP calculation of Bisphenol A. A QMRF report for the OPERA models predicting Fish Bioconcentration Factor is provided as Additional file 1.

**Fig. 5:**
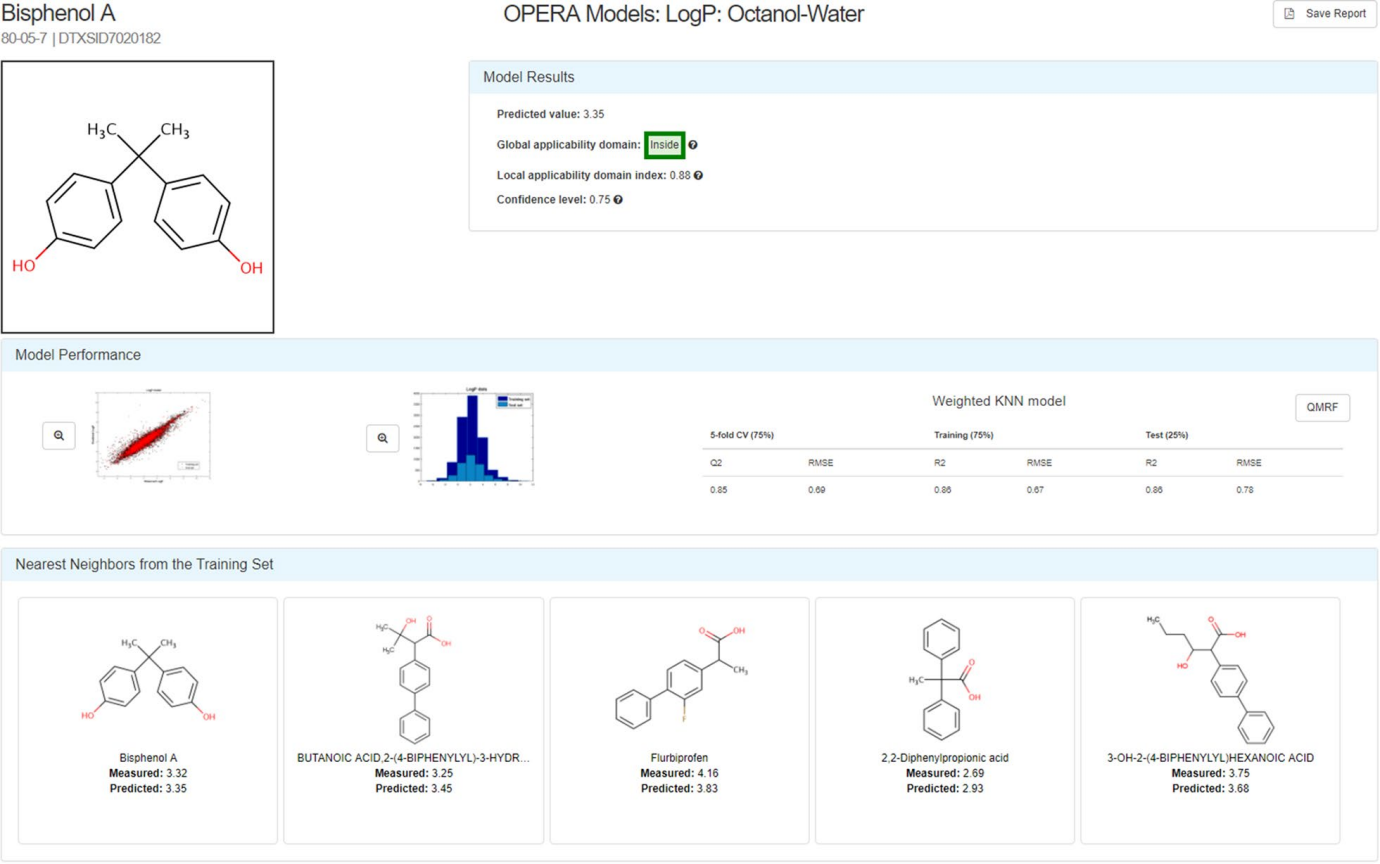
The OPERA Model Calculation Report for the logP calculation for Bisphenol A [79]. The green box containing the word “Inside” indicates that the prediction for this chemical is within the Global Applicability Domain for the model. The Model Results box displays both the local applicability domain index and the confidence level (both ranging from 0 to 1) for the prediction. The Model Performance block of the report provides a plot of the experimental versus predicted data for both the training and test data, as well as a graphic of the distribution of data values for the training and test data. The Weighted kNN model performance characteristics are listed for the 5-fold Cross-Validation and Training/Test 75%/25% splits. Up to 5 nearest neighbors from the training set are shown, along with the measured and predicted data for each
2. *EPA Toxicity Estimation Software Tool (TEST)* The Toxicity Estimation Software Tool (TEST) allows for the prediction of a series of physicochemical and toxicity endpoints using a variety of QSAR methodologies. TEST is available as installable Java applications for Windows, Mac and Linux [80] but has recently been ported to provide a set of web services. These services will be made publicly available at a later date (scheduled for Spring 2018), but for the current Dashboard release were used to perform batch predictions of available physicochemical properties. QMRF reports are not available for TEST Models. Calculation Reports will be available for all TEST endpoints in the future (scheduled for December 2017); an example is available for prediction of the viscosity of acetonitrile [81].
3. *ACD/Labs* ACD/Labs is a commercial software provider and markets the Percepta software for the prediction of physicochemical, ADME and toxicity data [82]. NCCT has licensed the Percepta software and uses all three modules listed above to populate internal databases. A subset of the ACD/Labs physicochemical prediction data has been made available for public release via the Dashboard, e.g., logP, boiling point (BP), and vapor pressure (VP). Neither QMRF reports nor Calculation Report details are available for ACD/Labs software predictions via the Dashboard.
4. *EPI Suite* The EPI (Estimation Programs Interface) Suite™ software is a standalone Windows-based suite of physicochemical property, environmental fate and ecotoxicity estimation programs developed by EPA and Syracuse Research Corp. (SRC) [83]. The EPI Suite predicted data in the Dashboard were obtained for a subset of the dashboard content using the batch processing features available from within the EPI Suite application. Web services for these estimation programs have recently been made available [84]. EPI Suite predictions will be made for all chemicals in the DSSTox database for which structures can be batch processed in the near future using these services. Neither QMRF reports nor Calculation Reports are available for EPI Suite predictions via the Dashboard.
5. *NICEATM models* The NICEATM models [85] were built using the same PHYSPROP open data used in the development of the OPERA models. Models were built for six physicochemical properties: logP, logS, BP, MP, logVP and log Bioconcentration Factor (BCF). QMRF reports are available for the NICEATM models, but Calculation Reports are not available.

### Environmental fate and transport

The environmental fate and transport tab contains experimental and predicted properties sourced from online databases or predicted using EPI Suite, NICEATM, TEST and OPERA models, as discussed in the previous section. Included are properties such as the adsorption coefficient, atmospheric hydroxylation rate, biodegradation half-life, fish biotransformation half-life, as well as parameters to assess bioaccumulation potential, such as bioaccumulation factors (BAF) and bioconcentration factors (BCF). The properties are predominantly predicted values derived using OPERA models. EPI Suite models also are available for predicting bioconcentration and bioaccumulation factors, as well as the adsorption coefficient, and TEST and NICEATM models are available for predicting BCF. Experimental values for fish biotransformation half-life, BAF and BCF, were taken from the curated PHYSPROP database.

### ToxValDB

As previously described, the ToxValDB database aggregates “toxicity values” of various types from a number of public data sources. These toxicity values consist of many different dose measures captured at either the study or chemical level, and include measures such as PODs, LOALs or LOAELs, NOALs or NOAELs, No effect or Low effect levels (NEL or LELs), cancer-related quantities (cancer slope factors, inhalation unit risk), and other derived quantities such as RfDs and EPA Regional Screening Levels [86].

**Fig. 6:**
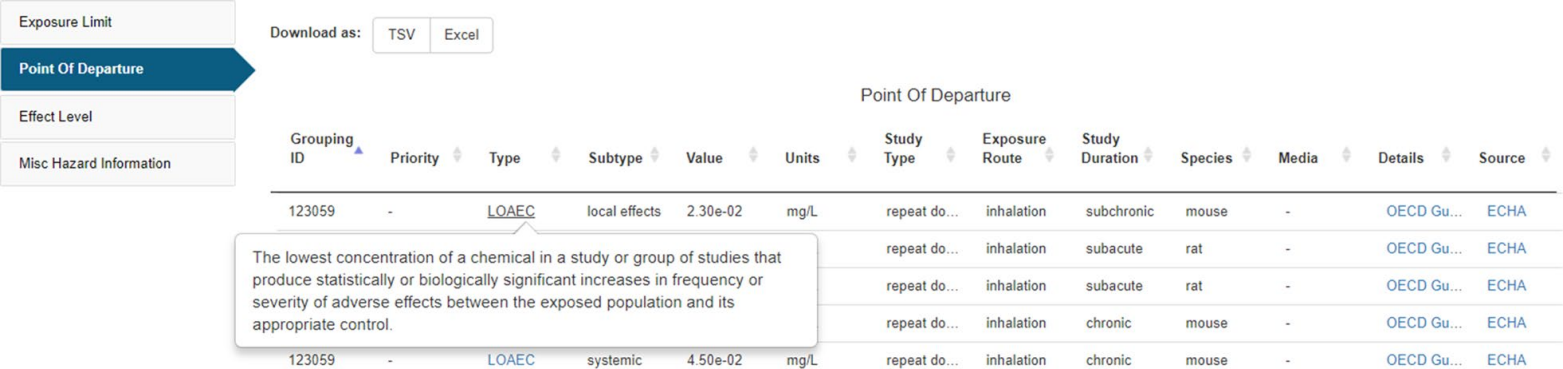
A subset of the ToxValDB data available for *N*-vinyl-2-pyrrolidone [87]. Note that the blue text, on hover, indicates either definition details on hover (as shown for the definition of the toxicity type LOAEC). The blue text further exists as a hyperlink to additional details that are displayed in a new browser window (e.g., for the last two columns in the ToxValDB table for Details and Source information)

The bulk of the information in ToxValDB was derived from systemic animal (mainly rodent) toxicity studies, including subchronic, chronic, reproductive and multigenerational reproductive studies. More detailed information, such as the data source reference, is also contained within the database and is viewable in the Dashboard by hovering over the source details (see Fig. 6).

### Absorption, distribution, metabolism, and excretion (ADME)

In vivo toxicokinetic (TK) data describing the ADME properties of chemicals as they pass through the body are unavailable for most chemicals [88]. However, estimates of toxicokinetics are necessary to extrapolate in vitro conditions (e.g., bioactive concentrations assessed in ToxCast assay) to the predicted real-world exposures (e.g., mg/kg bodyweight/day) that might correspond to those bioactive concentrations in tissues of humans or test animals [89]. To fill this data gap, in vitro methods have been used to characterize some chemical-specific aspects of TK for several hundreds of chemicals [88, 90].

The two primary chemical-specific ADME properties that are measured in vitro are plasma protein binding and metabolic clearance by pooled hepatocyte suspensions [88]. The former is used to calculate tissue partitioning and volume of distribution, while the latter measures are used to compute kinetic properties, including the dose half-life, steady state concentration, and the number of days needed to reach steady state [91]. The steady state concentration (Css, concentration at steady state given a 1 mg/kg/day oral dose), in turn, allows for simple in vitro to in vivo extrapolation estimation [88, 90]. All data and models used to derive TK properties have been made publically available [91] and predictions have been posted for 553 chemicals on the Dashboard. Since there are many more chemicals included on the Dashboard than have been characterized in vitro, QSAR models are being developed to predict these two key in vitro parameters [92, 93]. When confidence in the predictive ability of these models has been sufficiently demonstrated, the in silico predicted values will be integrated into the Dashboard along with the resultant estimates of volume of distribution, half-life and steady state concentration.

### Exposure

The Exposure tab contains a series of sub-tabs providing access to the following types of data, when available, for a particular chemical: (1) Product and Use Categories; (2) Chemical Weight Fraction; (3) Functional Use; (4) Monitoring Data; and, (5) Exposure Predictions. The first three are factors that have been found to be important indicators of exposure likelihood and are drawn directly from CPDat. The ‘Product and Use Categories’ tab for a particular chemical provides access to the Product Use Categories (PUCs) assigned to products where that chemical is an ingredient. In addition, the tab contains all CPCat use classes associated with that particular chemical [23]. The ‘Chemical Weight Fraction’ tab data is either directly extracted from the MSDS sheet data, when available [24], or is estimated based on the ordering of the ingredient list and the rules regarding how ingredient labels are created [94]. The ‘Functional Use’ data is either based on reported data or predicted by functional use QSAR models built on the harmonized functional use categories derived from reported uses [63]. The functional role a chemical may have in a product, in turn, can inform the concentrations that are likely to be observed.

The remaining two tabs contain inferred and predicted chemical exposures. ‘Monitoring Data’ provides the chemical exposures derived based on National Health and Nutrition Examination Survey (NHANES) [95] biomonitoring data collected by the U.S. Centers for Disease Control and Prevention. NHANES is a rolling survey covering roughly ten thousand individuals every 2 years, and biological samples (urine, blood, and plasma) are analyzed for a variety of biomarkers of chemical exposure. Although only ~ 100 chemical exposure rates have been inferred directly from NHANES, these inferred exposure rates have served as a training set for the development of consensus model ‘Exposure Predictions’. EPA’s Systematic Empirical Evaluation of Models (SEEM) framework allows prediction of exposure rates for thousands of chemicals [34], although these are significantly more uncertain than the exposure rates for chemicals directly inferred from NHANES.

### Bioassays

The Bioassays tab contains two sub-tabs, one that displays Toxcast and Tox21 HTS data, if available, and the other that displays available PubChem Bioassay Data [96]. The PubChem data are retrieved in real-time using a PubChem widget [97] which accesses the PubChem API, displaying bioassay data associated with the chemical in question. The PubChem data can be further refined and analyzed using the built-in capabilities of the widget, and the data can be downloaded as a CSV (comma separated values) file. As will be described later, DSSTox substances and associated structure content have been deposited in PubChem through associated PubChem IDs.

**Fig. 7:**
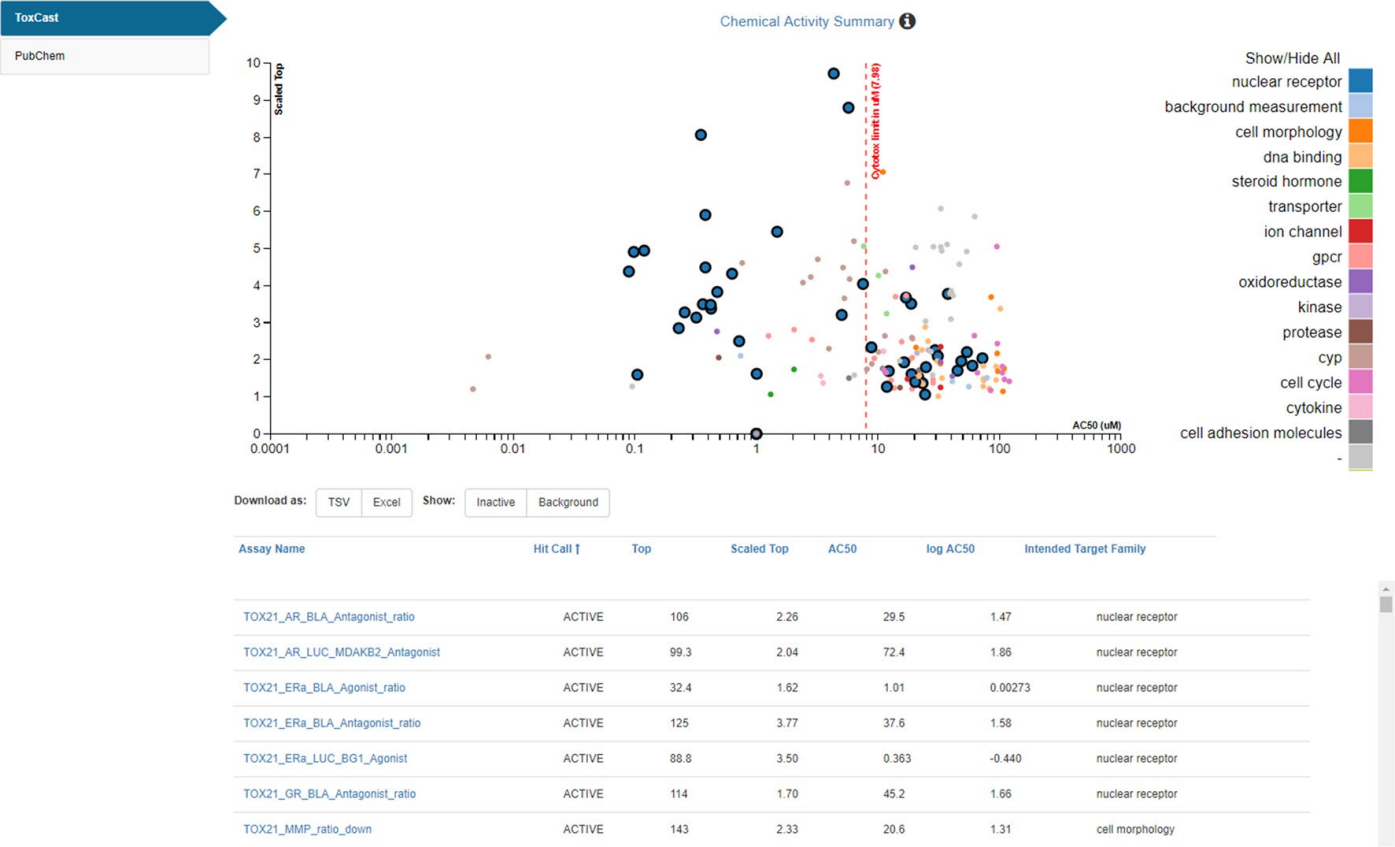
The visualization of Active hit-calls for Bisphenol A under the ToxCast subtab [98]. When hovering over a particular target class on the legend (right-hand side), the associated values are highlighted on the plot. The red-dotted vertical line indicates the Cytotoxicity Limit (i.e., the dose at which cytotoxicity is observed). The assay data table below the graphic shows Active hit calls by default but the Inactive and Background data can be included in the table by selecting the toggle buttons above the table. The data can be downloaded as TSV (tab-separated values) or Excel files

The ToxCast/Tox21 HTS summarized results for a tested chemical can now be viewed directly through the Dashboard. A graphical plot is displayed in the panel showing modeled AC50 (concentration that elicits a 50% response) values for ACTIVE hit calls, which are color-coded according to different target classes (e.g. steroid hormone, nuclear receptor, GPCR, and others). Hovering over a specific target (right hand side of the visualization widget) highlights data for that particular target (see Fig. 7).

The scaled activity values shown on the graph are calculated by dividing the response values by the activity cutoff, thereby enabling activity comparisons across assay endpoints. The data displayed are from multi-concentration experiments only. A previously published dashboard application, the Toxcast Dashboard [99], also provides full access to single concentration assay data in the list of ‘tested’ assays endpoints if multi-concentration data are unavailable.

**Fig. 8:**
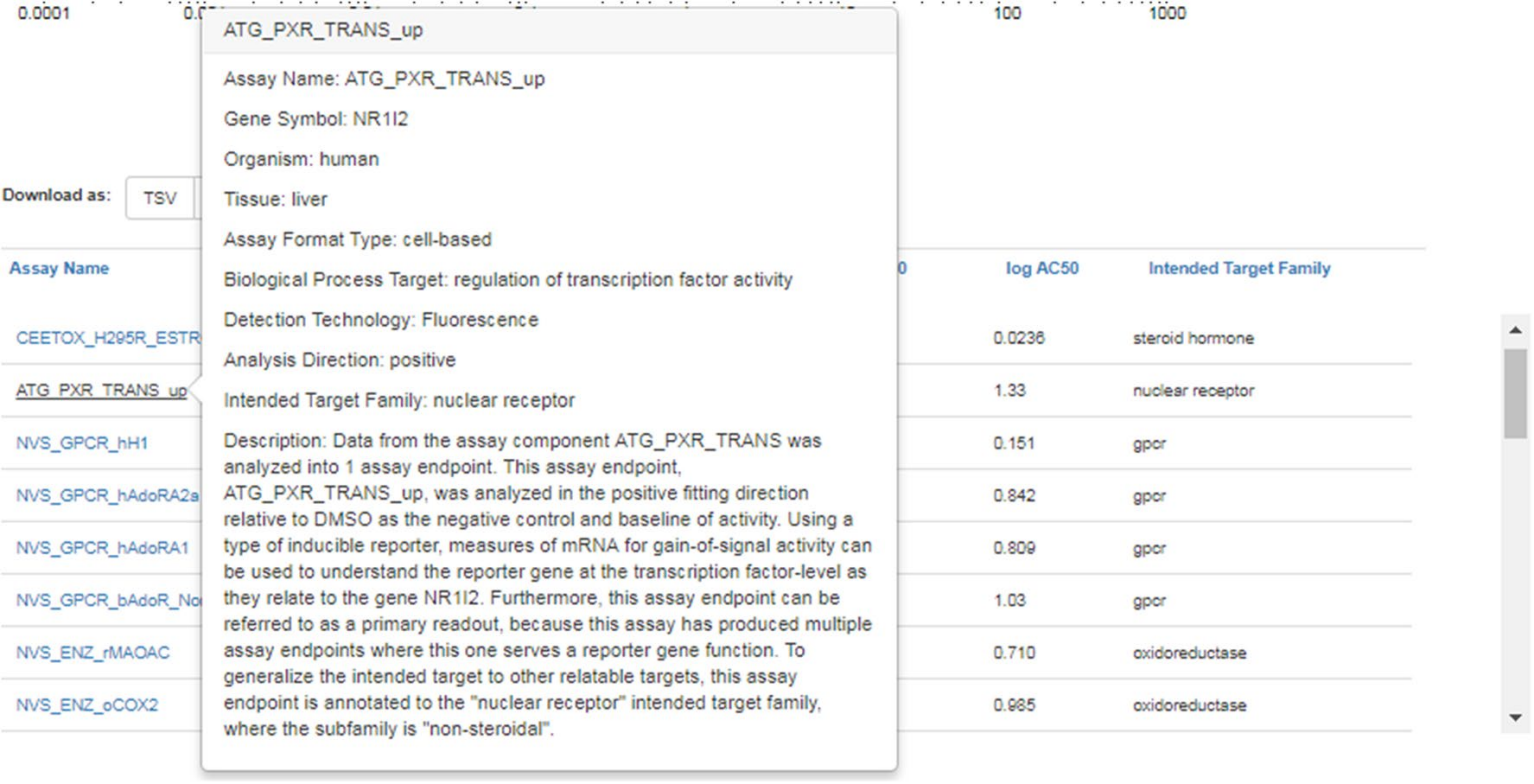
Information regarding a particular in vitro screening assay is accessed by hovering over the name of the assay to display details in a modal (user interaction) window

The table below the bioassay plot lists assays and associated Top, Scaled Top, AC50 and logAC50 activity values that have been measured for the chemical in question. The default table display includes only Active hit calls, but Background and Inactive hit calls for other assays can be toggled on/off. Hovering over the Assay Name lists the details of a particular assay in terms of organism, tissue type, measurement technology and other details (see Fig. 8). The assay data associated with a particular chemical can be downloaded in both TSV and Excel data format. Raw, normalized, and interpreted single concentration data are also available from the freely downloadable MySQL version of the InVitroDB database [100].

### Similar molecules

The similar molecules tab shows the results of a structural similarity search, underpinned by a Tanimoto similarity calculated using the Bingo Molecular Search Cartridge (with the associated Indigo fingerprints) [71]. The search displays up to 50 of the top-most similar molecules above a Tanimoto similarity metric of 0.8. The view also displays a selection of experimental and predicted chemical properties to help illustrate the consistency and concordance of these attributes within the identified set of structurally related molecules.

### Synonyms

The synonyms tab contains a compiled list of systematic and non-systematic names, trade names, trivial names, CASRNs, Beilstein IDs and U.S. Food and Drug Administration (FDA) registry numbers. Synonyms were collected from public sites (such as PubChem, ChemSpider, ChEMBL, ChemIDPlus, and ACToR), migrated from approved source lists, entered by members of the DSSTox curation team, and generated using systematic nomenclature software. All data are held within the DSSTox database and are used to generate a synonym lookup file that is consulted by text-based chemical name searches. The data are listed in the Dashboard using three font styles: **bold** for Valid Synonyms (manually curated by the team or algorithmically generated by systematic naming software), *italicized* for Good Synonyms (as a result of seeing consensus across a series of public databases), and normal font for Other Synonyms. The synonyms table additionally can include other CASRN (deleted or alternate) publicly associated with the substance, but not assigned by DSSTox curators as the unique “Active” CASRN, so that searches can return appropriate results.

## Literature

The literature tab provides access to various types of literature associated with a chemical compound, both as searches (against Google Scholar (GS) and PubMed) and via direct linking (to PubChem Articles and PubChem Patents), and as embedded PDF files accessed from EPA websites.

**Fig. 9:**
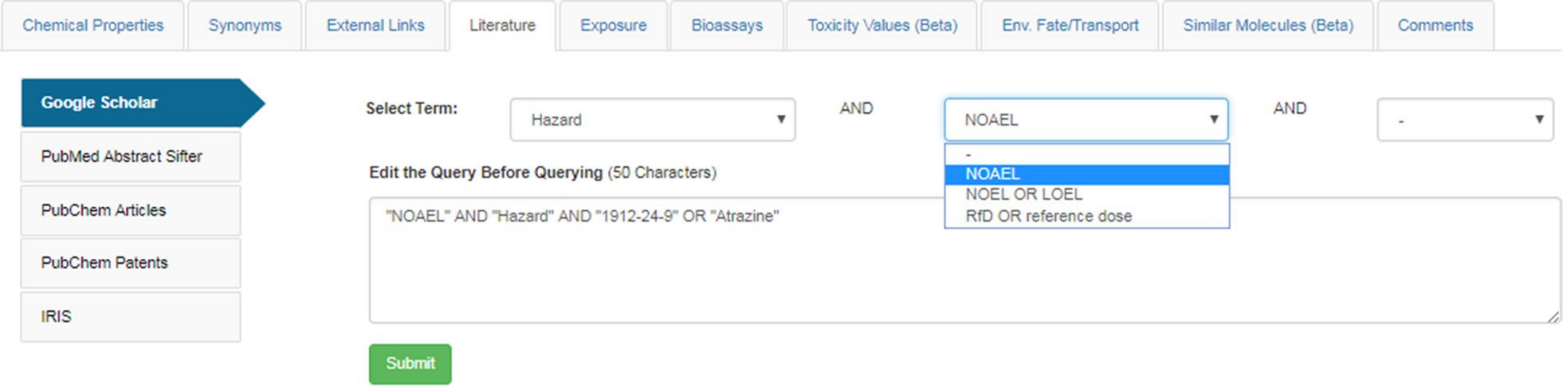
The Google Scholar search interface in the Dashboard. A term of interest is selected from the pulldown list and secondary and tertiary terms, if available, can be chosen. In this case a Google Scholar search for information regarding atrazine as a Hazard with available NOAEL (no observed adverse effect level) data produces a simple query that is passed to Google Scholar when the Submit button is clicked

The GS search integration assembles a search query to pass to GS that includes the associated CASRN and Preferred Name for the chemical, along with a nested set of queries that can be selected by the user. For example, the selection of Hazard (from a set including Fate and Transport, Metabolism, Exposure, Male Reproduction, and others) produces a secondary set of nested queries (including NOAEL, NOEL OR LOEL, RfD or Reference Dose) for the user to select (see Fig. 9).

**Fig. 10:**
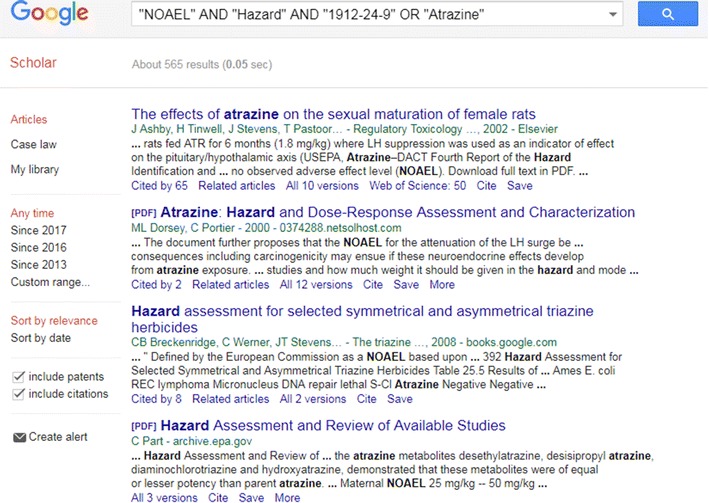
The results set obtained by passing the query defined in Fig. 9 to Google Scholar [101]

For example, to retrieve literature references for the chemical ‘Atrazine’ a selection of the terms from the pulldown menus produces an associated search query of ““NOAEL” AND “Hazard” AND “1912-24-9” OR “Atrazine””, producing ~ 600 results in the GS search that the user can browse, further filter, or download (see Fig. 10).

Whereas users could utilize the GS search query interface directly, access via the Dashboard reduces the barrier to such a search by providing the associated CASRN, preferred name and nested query sets as a starting point. The user can add additional query terms either into the search box in the Dashboard or in the GS search interface. Since the GS search is text-based (rather than structure-based), CASRN mixtures and categories of chemicals, such as polychlorinated biphenyls (PCBs) [102], can be searched to retrieve potentially useful results [103].

**Fig. 11:**
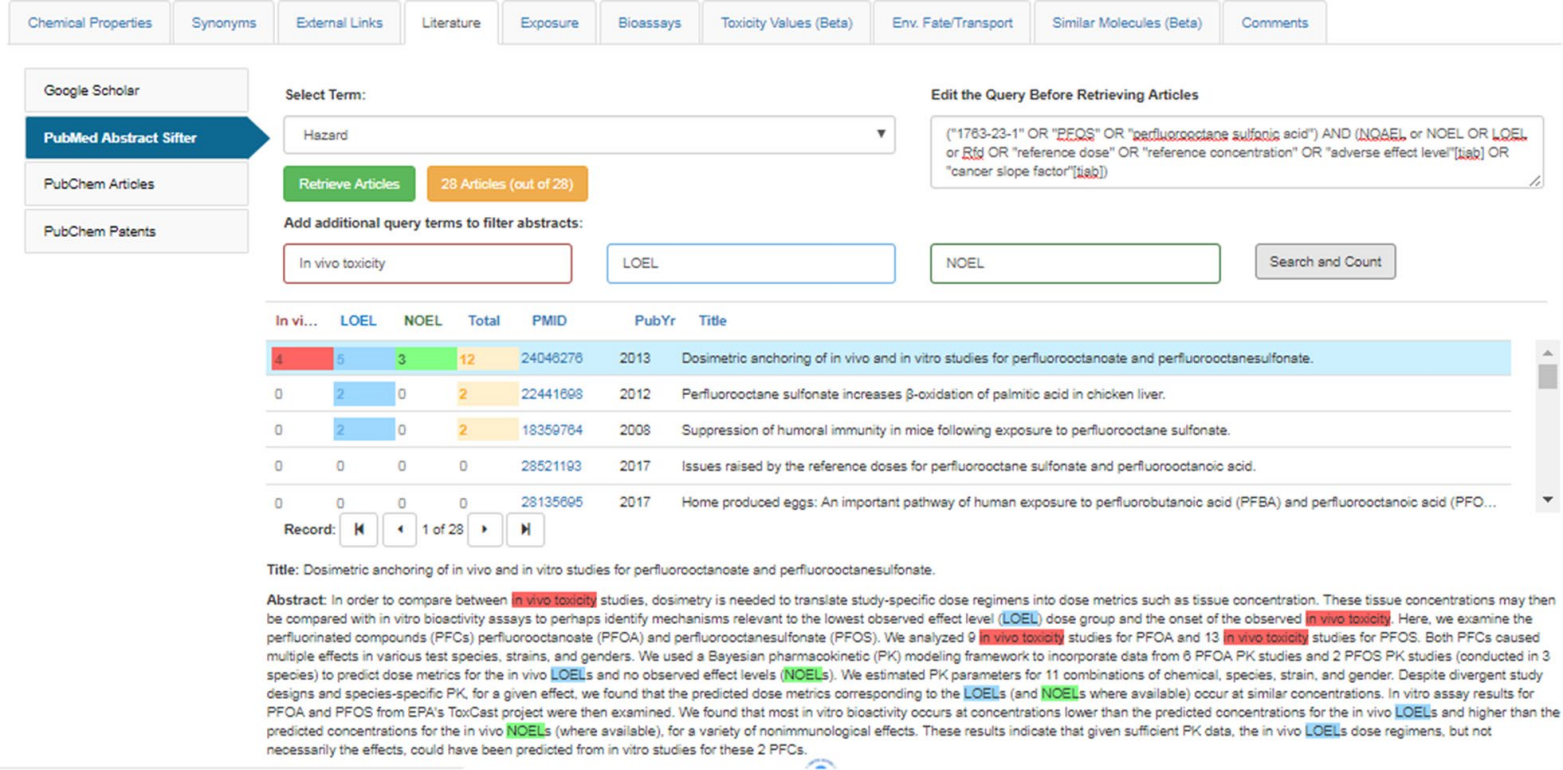
The PubMed Abstract Sifter interface. Following selection of a term to include in a MeSH-based query including the CASRN, chemical name and MeSH name a query is run against the PubMed services to return up to a maximum of 1000 article abstracts. The list of abstracts is downloaded to the Dashboard and can be filtered using up to 3 additional query terms that are highlighted, in color, on the abstract. The interface allows for rank-ordering using the query terms and click-through to the PubMed article using a hyperlinked PubMed ID (PMID)

The PubMed Abstract Sifter search capability surfaced in the Dashboard is a limited implementation of work reported by Baker et al. [104] and utilizes a MeSH-based [105] query against the PubMed services [106]. The Abstract Sifter employs a similar search query interface to that provided by the GS search, and is layered upon the DSSTox database, so is based on using the more highly curated CASRN and preferred name for a chemical substance, in conjunction with the more broadly inclusive MeSH-name for the chemical. User-generated nested queries are not available in this implementation; rather, Sifter queries are focused on pre-loaded terms of interest to toxicology and exposure. Selection of a query term, for example Hazard, extends the chemical identifier list with a pre-generated MeSH query associated with the term of interest. For a substance such as PFOS [107], a Hazard based query would produce (“1763-23-1” OR “PFOS” OR “perfluorooctane sulfonic acid”) AND (NOAEL OR NOEL OR LOEL OR Rfd OR “reference dose” OR “reference concentration” OR “adverse effect level”[tiab] OR “cancer slope factor”[tiab]) as input. Whereas a GS search query navigates the Dashboard user to results on the GS site, the Sifter accesses external PubMed web services and returns an abstract count directly to the Dashboard interface. If a large number of results are retrieved, the user can refine the query by adding additional filter terms or download the set to the off-line Sifter application. Typically, the number of search results is far fewer; for the example of PFOS listed above, 28 abstracts are downloaded into the web interface for further ‘sifting’. As shown in Fig. 11, the titles and abstracts can be further filtered in the Dashboard interface by adding query terms into the three boxes shown (e.g., in vivo toxicity, LOEL and NOEL). Clicking the button “Search and Count” filters and color highlights the query terms in the interface results view. Each column can be sorted based on rank (i.e., relevance of results to the selected query terms). Clicking on the PubMed Identifier (PMID) provides a hyperlink through to the abstract (or the full article in the case of an Open Access article) on the PubMed website. Again, a trained user could reproduce this query on the PubMed site, independently, but the integration of Abstract Sifter via the Dashboard interface greatly facilitates these types of searches by pre-formulating MESH queries for the user and returning results to the Dashboard.

The PubChem Article and Patent sub-tabs integrate the associated PubChem Widgets (see sections 5 and 6 in [97] and display (PubChem) depositor-provided PubMed citations and Patent Identifiers for the DSSTox chemical in question in embedded table views. Again, since all DSSTox substances and associated structure content have been deposited in PubChem, direct linkages to PubChem content are available through PubChem identifiers (CIDs).

EPA’s IRIS [60] and PPRTV [61] reports are integrated into the Dashboard as PDF files. The integration is based on list curated mappings between the chemicals in the DSSTox database and publically available documents. For example, for acrylamide, the IRIS document number 286 is mapped to the substance in DSSTox and the associated URL is used to load the PDF file into the interface using [108]. A similar approach is used to link through to PPRTV documents, again using the associated URL for the document.

Both the IRIS and PPRTV chemical lists are available via the dashboard. At the time of writing, the IRIS list includes 510 distinct substances [109] and the PPRTV list includes 403 substances [110]. Each list can be downloaded with DSSTox standard chemical identifiers (e.g., DTXSID, CASRN, Preferred name, SMILES, etc.) in Excel and SDF formats from the list interface. As new IRIS or PPRTV records are released, the lists will be extended by adding new substance mappings.

### External links

An external links tab provides integrated searches or links to ~ 70 online external resources and databases. Some of these are EPA resources, but the vast majority are non-agency public resources. Links are based on a simple URL-based approach, where a site is accessed using one of the identifiers associated with a chemical as the linking parameter. Identifiers that can be used include one or more of the associated CASRNs, the preferred name, the InChIKey or SMILES string, or a source parameter registered into the underlying DSSTox database through the list-mapping curation process. The resources that are presently available from the External Links tab are listed in Additional file 2 and include several large public resources of analytical spectra and properties, as well as toxicity data.

Specific examples of external links are highlighted below using atrazine [72]. Web resources such as the National Institute of Standards & Technology (NIST) Webbook and the National Environmental Methods Index (NEMI) are accessed using the **bolded** CASRN in the query URL strings: NIST Webbook http://webbook.nist.gov/cgi/cbook.cgi?ID=C**1912-24-9**&Mask=200#Mass-Spec [111] and NEMI https://www.nemi.gov/methods/analyte_results/?media_name=&source=&instrumentation=&analyte_code=**1912-24-9** [112 ]. Springer Materials and ChemRTP Predictor use the InChIKeys in the respective query URLs: http://materials.springer.com/search?searchTerm=MXWJVTOOROXGIU-UHFFFAOYSA-N [113] and http://www.chemrtp.com/chemical-info.ce?ID=MXWJVTOOROXGIU-UHFFFAOYSA-N [114], respectively. In certain cases, chemical sets have been mapped into the underlying DSSTox data using their own identifiers to allow direct hyperlinking. These include ECHA Infocards (https://echa.europa.eu/substance-information/-/substanceinfo/**100.016.017**) [115], the mzCloud mass spectral database (https://www.mzcloud.org/compound/Reference/**42**) [116], the Comparative Toxicogenomics Database (http://ctdbase.org/detail.go?type=chem&acc=**D001280**) [117] and NIOSH Chemical Safety Cards (https://www.cdc.gov/niosh/ipcsneng/**neng0099**.html) [118]. In all cases the resource identifier is bolded in the URL string. The value of these resources to Dashboard users justifies the ongoing maintenance of the mappings that is required to support the link-outs.

Adding new external links to the Dashboard is a relatively simple process that does not require direct coding in the system but, rather, requires only a few text entries into the Administration Panel (see below). When it is known that a chemical is either not indexed on an external resource, or has no data on that resource, an attempt is made to convey this by removing the hyperlink and “greying out” the text in the Dashboard. This information is not available for all sites, however; additionally, ongoing review of links to external resources to add new links or prevent what is known as “link rot” (i.e., links removed or changed by external sites such that the original link no longer works) is carried out on a quarterly basis.

### Comments

Crowdsourced curation of data is increasingly becoming a mainstream approach to improving data quality for online resources. Notable examples for the curation of chemistry data specifically include Wikipedia [119] and ChemSpider [120]. With the DSSTox dataset containing 760,000 chemical substances, and with the growing volumes of associated data for each chemical, the gathering of feedback from users as they navigate through the data is a helpful and efficient approach to elevate data quality. For every chemical page, a “Submit Comment” button allows a user to provide feedback regarding the data shown in the Dashboard. Almost 200 public comments have been submitted as of November 2017 [67]. The majority of these report mis-mappings of chemical names and chemical structure depictions. This application allows the Dashboard administrators to address the comments, make corrections if needed, email the user directly with the response, and the responses are public for all to view and review. The vast majority of comments received to date have been addressed, and the fixes have been incorporated into later releases of data.

### Advanced search

**Fig. 12:**
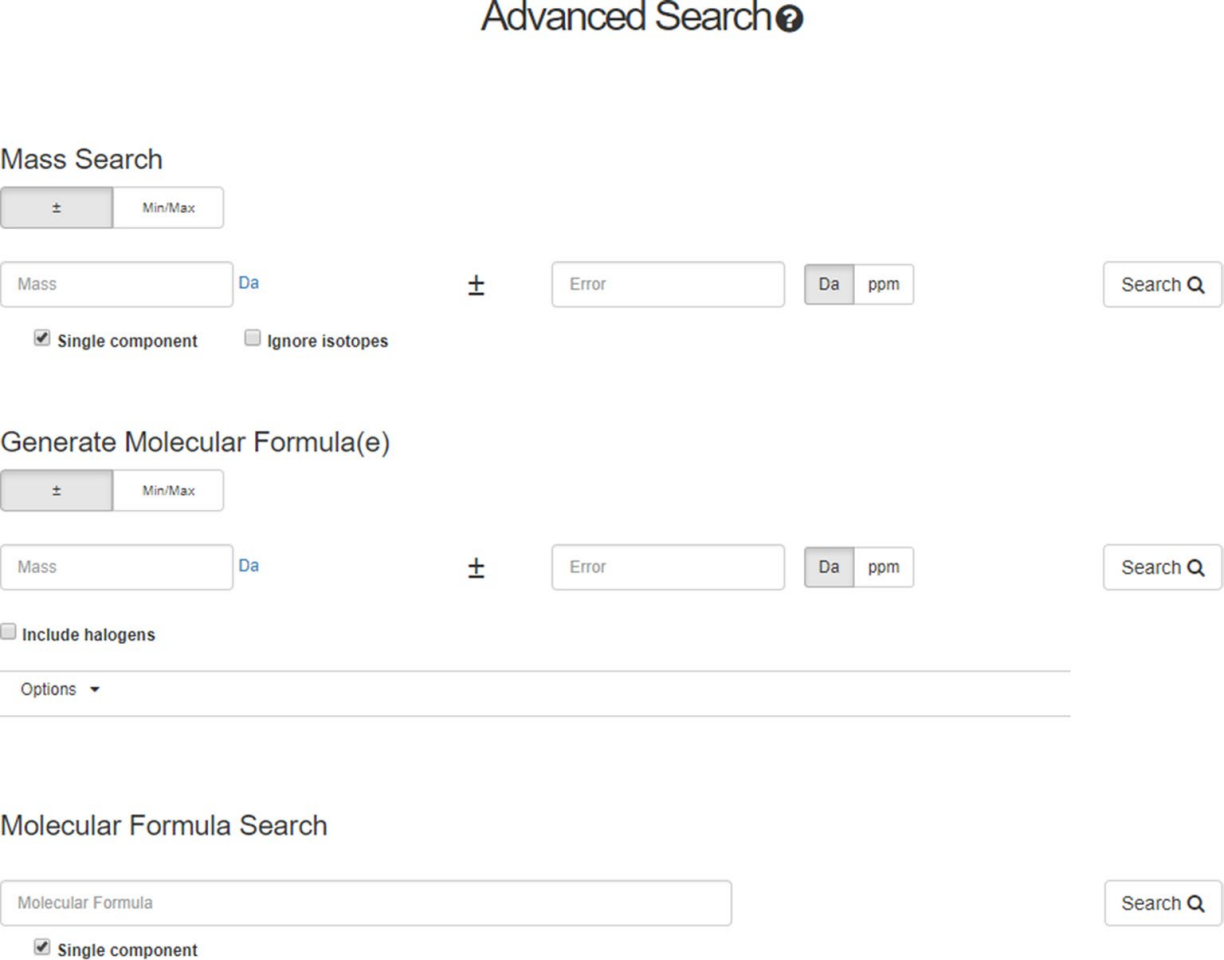
The Advanced Search allows searches based on Mass and Molecular Formulae (including a single/multiple component filter). It is also possible to enter a mass (with error) and generate molecular formulae and perform a search across all dashboard content. For example, a search for a mass of 300.1220 ± 5 ppm produces 536 formulae with only four of them mapped to chemicals in the database [122]

An advanced search feature on the Dashboard (Fig. 12) allows for mass and molecular formula searching, and molecular formula generation (based on a mass input). The search operations are explained in detail in the Help manual [121].

The formula and mass-based searches have been specifically designed to support non-targeted mass spectrometry research conducted within the EPA [123, 124] as well as to support global needs for this type of informatics resource. This advanced search capability is increasingly used by collaborators involved in the ENTACT project, an EPA-led international collaboration involving ~ 25 laboratories and focusing on the evaluation and refinement of non-targeted analysis methods [125]. The Dashboard application developed for this purpose is discussed in the Applications section of this paper (vide infra).

### Batch search

**Fig. 13:**
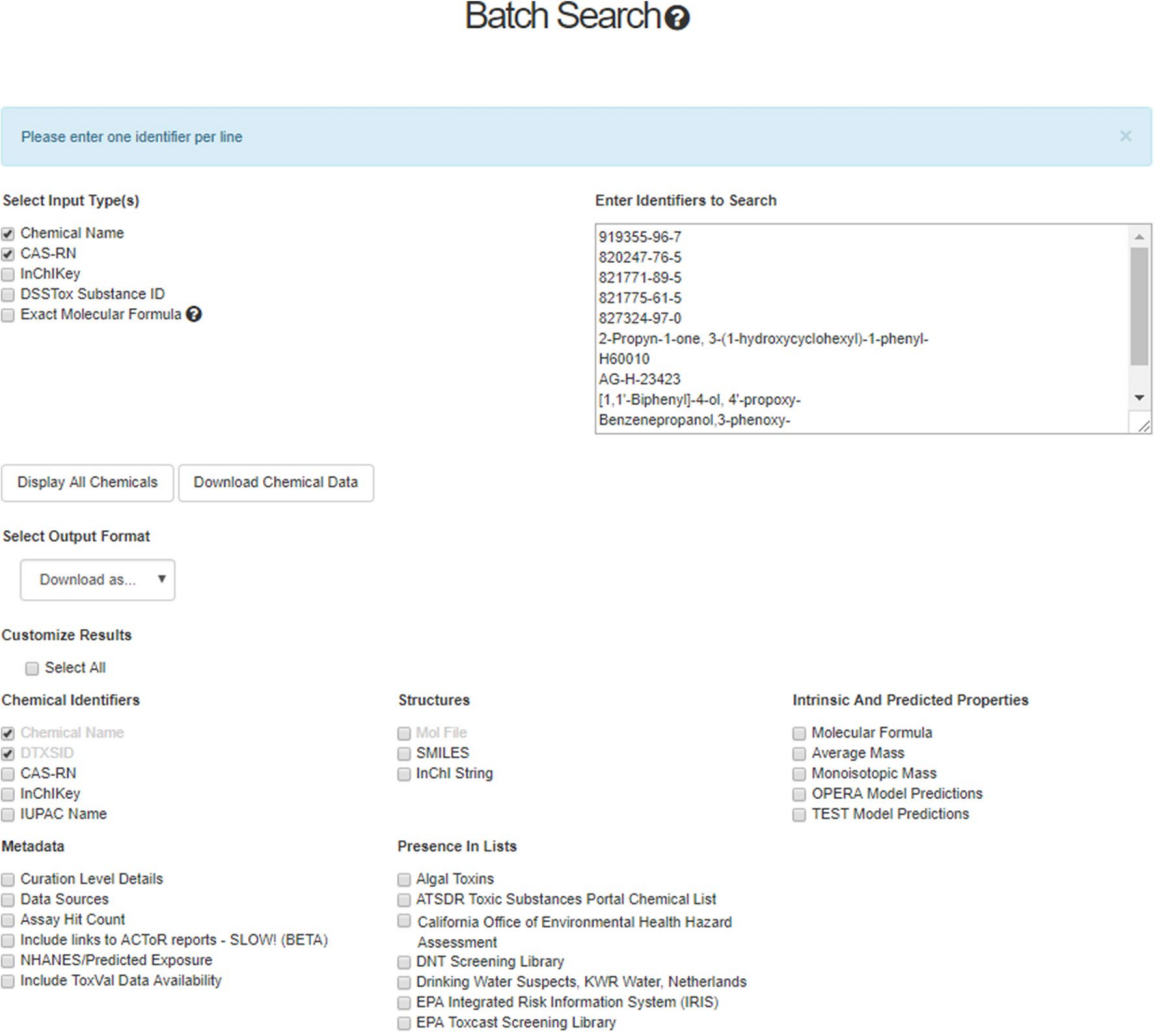
The Batch Search allows for searching the database using inputs of Chemical Name, CASRN, InChIKey, DTXSID and Exact Molecular Formulae. The user can display all chemicals or download the resulting file as a tab-separated value (TSV file), an Excel spreadsheet or an SDF file. The user can choose what to include in the download file and can select from a series of chemical identifiers, structure forms and chemical properties (including OPERA and TEST predictions). Metadata can include ToxCast assay hit count and the availability of Toxicity Values

A batch search (Fig. 13) feature allows users to input lists of chemical identifiers (hundreds to thousands) to perform a customized list mapping to DSSTox content and associated data. This feature delivers standard DSSTox identifier content (including structures as mol or SMILES), in addition to valuable initial list curation feedback to the user via internal mapping functions. For instance, invalid CASRN (failing the CASRN checksum [126]) are flagged, deleted or alternate CASRN are rerouted to the active CASRN, “No Hits” are indicated, and valid synonym mappings are used to retrieve associated substance matches that might have non-matching source IDs. The user can further direct a batch search to download selected data and metadata associated with the successfully mapped portion of the original chemical list. The accepted inputs include chemical names, CASRNs, InChIKeys, DTXSIDs and Exact Molecular Formula, and these can be used to retrieve formulae, masses, DTXSIDs, and other data related to chemical bioactivity and exposure.

### Lists

Another feature of the Dashboard is the chemical lists. These lists provide access to an aggregate of chemicals associated with a project, publication, source database, or other collections. An index page listing a set of public DSSTox registered chemical lists is accessed via the top banner menu “Lists” link on the Dashboard [127]. Each registered list is accompanied by the list title, the number of associated chemicals in the list, and a short summary. At the time of writing, almost 40 lists were available ranging from a small algal toxin list containing 54 compounds [128] to the much larger Tox21 Screening Library containing 8947 chemicals [129]. A more detailed list description, as well as a tabular view of the chemical structures included in the list, are accessed by clicking on the list name. Lists can be assembled in two ways: through a defined list curation process that registers the source list in the underlying DSSTox database, or at the application level. In the latter case a list is generated by initially mapping to DTXSID content, where possible, but the data are not yet fully curated or internally registered as a DSSTox list. The list curation process to fully register a list in DSSTox involves not only initial mapping of source IDs to DTXSIDs, where possible, but also identification of “No Hits” and delineation of partial or tentative source-substance ID agreement. An example would be where a list containing CASRNs and Chemical Names are registered and the CASRNs agree but the names conflict. Each of these cases must be resolved by a DSSTox curator prior to the substance being fully registered in the list. Due to the large size and uncurated content of many public lists containing large numbers of conflicted ID records (observed in, for example, in TSCA, ACToR, CPDat, and PubChem), the current strategy is to internally store all source IDs and curation notes, and auto-register as much of the list as will cleanly map to DSSTox substances, while the remainder of the list is prioritized for more complete curation at a later time based on its importance to EPA programs.

### Web API

The Dashboard utilizes a number of existing web services based on ACToR [70]. These RESTFul services provide data in HTML, JSON, XML, PDF and Excel formats. At the time of writing, a number of new microservices and an associated API are under development to provide access to data and search results for integration into third party applications. An early example of the impending services is presently in alpha testing and provides access to the TEST predictions for a number of endpoints, an example being water solubility prediction [130].

### Navigation assistance and help manual

**Fig. 14:**
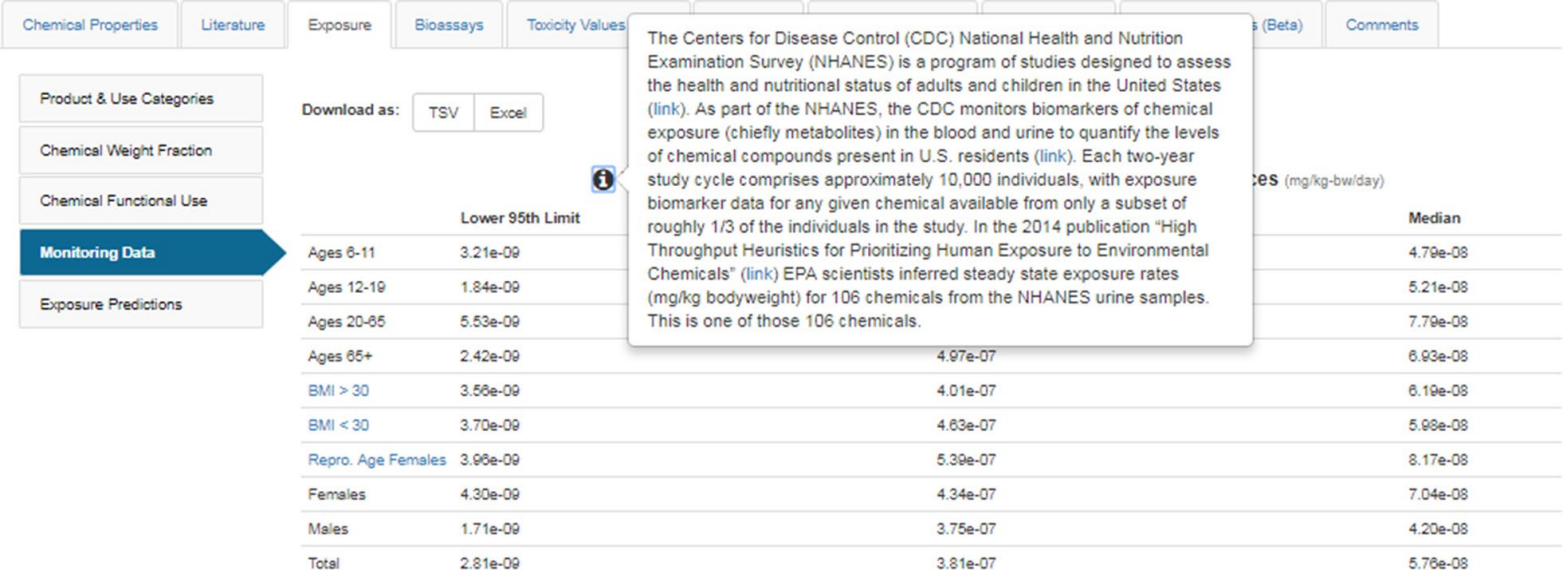
Help and Informational icons are provided across the Dashboard to inform users about particular functionality and data. This includes linking to relevant research papers as shown for the modeling of the NHANES modeling data

The Dashboard delivers access to multiple data types and resources, integrated into a single application. Nevertheless, certain types of data are unique to the application and naïve users will not necessarily be aware that the various data are available. A Help and information text annotation layer has been included so that additional details regarding the navigation of the Dashboard are available for the user. For example, under the Monitoring Data side tab, users may be unfamiliar with the “NHANES” data displayed in the Dashboard [131]. The informational icon (‘i’), when clicked, displays a detailed hover description that includes links to publications and related websites (see Fig. 14); in addition, the NHANES acronym in the title is hyperlinked to the source website. Adding new help or informational text to various parts of the application is managed through an Administration Panel using simple text entry boxes.

Users have previously left feedback on the site via the Contact Page [132] asking for details about particular data. An administrator can quickly respond with an email pointing them to an updated Help text on the Dashboard, answering their question and enhancing the application for the general community. A general Help manual for the Dashboard is also available [121] and we have initiated a project to integrate instructional videos into the dashboard (for example on the Advanced Search page a link to a video posted to YouTube is included [133]).

### Data downloads

The Dashboard not only allows access to a series of databases, but also allows downloading of data contained within those databases. A downloads page provides access to a number of pre-generated data slices [134]. The list currently includes: (1) a file mapping DTXSID and PubChem identifiers; (2) DTXSID identifiers mapped to CASRNs and chemical names; (3) a zip file containing a number of SDF files with the structure and associated DTXCID, DTXSID, Dashboard URL, associated synonyms, and DSSTox Quality Control Level details; and (4) curated physicochemical data underpinning the OPERA models and including the KNIME workflows used to prepare the data for QSAR analysis (e.g., deduplicating, desalting, structure normalization, tautomer recognition, etc.) [27]. These files are available as CC-Zero licensed data files from a FigShare page associated with NCCT (http://epa.figshare.com).

Some download datasets result from the registration of the DSSTox data collection into third party databases. DTXSID datasets mapped to identifiers such as PubChem CIDs can make registration into other databases using CIDs much easier. The DSSTOX mapping file, containing mappings between DTXSIDs and the associated InChI Strings and InChIKeys, greatly facilitated registration into UniChem [135], whereas the DSSTox SDF file made both PubChem (PubChem [136] and ChemSpider registration simple [137].

### Administration panel

The Administration Panel (admin panel) provides EPA developers with facile control of the Dashboard for a number of important functions related to informational help and notifications, responding to crowdsourced comments related to particular chemicals, responding to Site Feedback, and updating the Latest News segments displayed at the bottom of the home page. The admin panel also allows for the addition of new searches to both the Google Scholar and Pubmed Abstract Sifter tabs, the addition and maintenance of external links, and controlling information displayed on hovers defining chemical property sources. This level of administrative control, allowing additions in content while the Dashboard is in production, provides the ability to quickly respond to user feedback, add additional help comments, and incorporate new external links, new types of literature searches, etc.

## Applications of the Dashboard

The Dashboard delivers chemistry content linked to a series of data streams via a web-based interface that allows searches for content associated with single chemicals or batches of chemicals. Due to the integrated content, the Dashboard can be used to answer many different types questions, such as: (1) What is the structure of chemical X? (2) Is my query chemical contained in EPA’s ToxCast inventory? or the larger Tox21 inventory?; (3) What is the current full list of chemicals for which ToxCast data has been generated?; (4) For my list of 1000 CASRNs (or chemical names), are ToxCast in vitro bioassay data, in vivo toxicity data, and/or exposure prediction data available?; (5) For my list of 2000 chemical names, can the Dashboard provide predicted physicochemical and environmental fate and transport data?; (6) What products contain my query chemical and with what weight fractions?; (7) What literature abstracts are available linking my query chemical to the term “hazard”? The Dashboard can provide data that will inform the answers to these questions.

An example of how the Dashboard can help with chemical structure identification analyses is in the area of Mass Spectrometry (MS) and Non-Targeted Analysis (NTA). The use of NTA is increasingly being employed in environmental research to gather information on the real-world exposures to a broad range of chemicals potentially present in media such as wastewater [138], water [124, 139], dust [123], sediment and others. The goal of NTA in environmental research is not to attempt to confirm the presence of particular chemicals using standards, but rather to identify, with as much certainty as possible, the broadest range of chemicals detectable. Hence, NTA studies require cohesive workflows for candidate structure identification and prioritization [140], as well as large, accurately curated reference libraries of chemicals specific to the domain of environmental chemistry, such as provided by the DSSTox database [123, 141]. The Dashboard has been augmented with mass-search capabilities that make it a valuable resource for the NTA research community. Search functionality within the Dashboard enables users to perform queries based on a single monoisotopic mass or molecular formula (via the Advanced Search screen) or batches of many molecular formulae (via the Batch Search Screen). Mass and formula(e) searches of unidentified chemicals observed in NTA return not only candidate chemical structures, but also the uniquely linked substances and associated IDs, based on the search criteria. By rank-ordering the number of data sources of the returned results list, the most likely candidate structures are prioritized and returned to the user [142]. A recent example is the use of data downloads from the dashboard (vide supra) used as a source of candidate structures and as a suspect list within MetFrag [143, 144].

A Dashboard feature important for NTA is the advanced searching that includes “MS-Ready” structures that are desalted, desolvated, mixture-separated, and absent of stereochemistry to match to the neutral form of a chemical [145] observed by an analyst during NTA data processing [146]. Searching the formulae for a list of unknowns against MS-Ready structures links instrument observations to all forms of a structure contained within DSSTox (e.g., the neutral form and the hydrochloride salt or solvate of a structure). Further, additional data streams within the Dashboard (e.g., physicochemical properties, CPDat usage data, etc.) can be incorporated into identification schemes to inform the analyst of a candidate chemical’s method compatibility, use in commerce, likelihood of occurrence in a particular environmental media, etc. By combining advanced search functionality, MS-Ready structures, and rich data streams to increase certainty of identification in NTA, the Dashboard provides a valuable resource for the mass spectrometry NTA community.

**Fig. 15:**
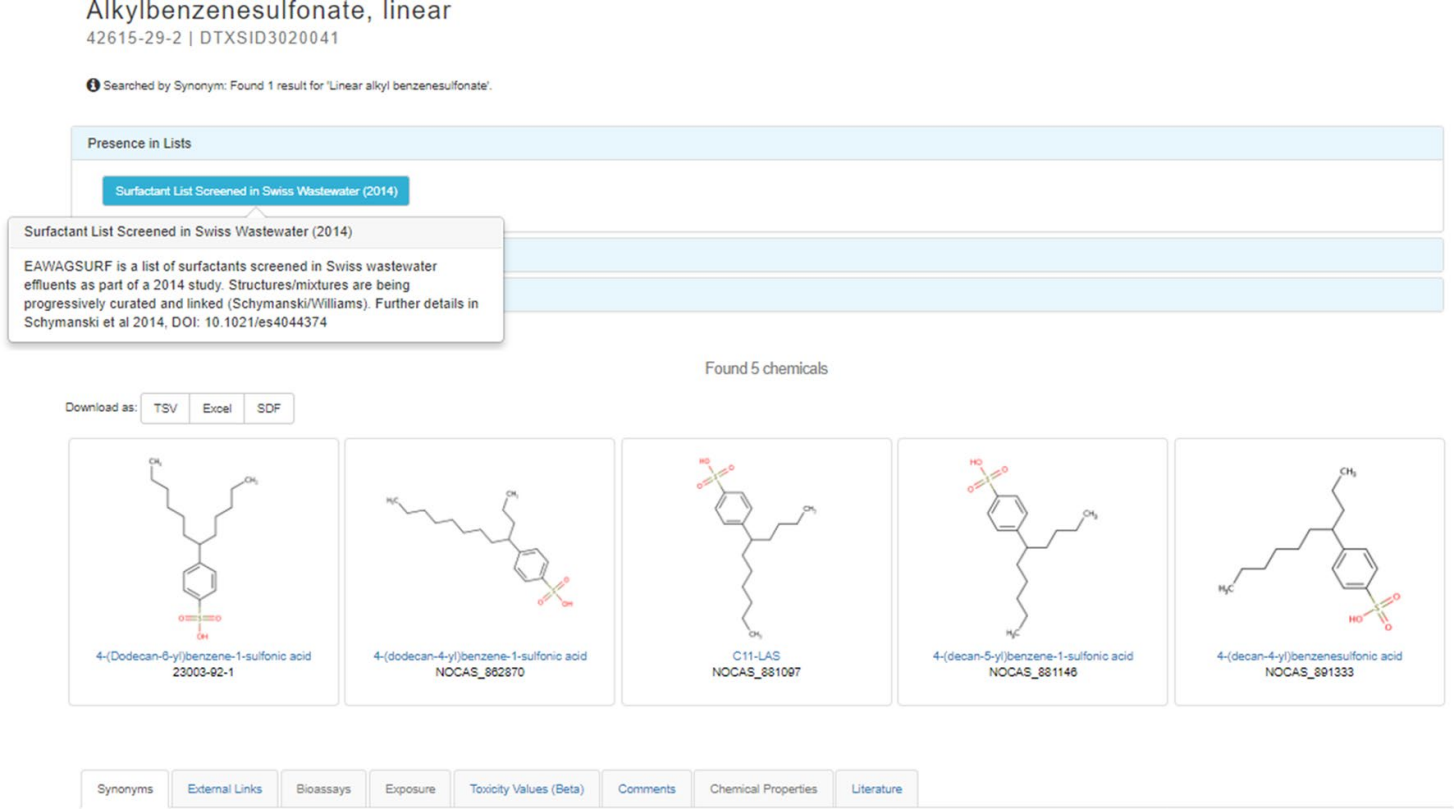
The Dashboard displays mappings between the linear alkylbenzenesulfonate surfactant (CASRN: 42615-29-2) and the mappings to five related chemicals. Notice the “Presence in Lists” accordion has the “Surfactant List Screened in Swiss Wastewater” identified and the hover detail describes where the data are extracted as a publication and associated DOI

Cheminformatics support for “UVCB chemicals”, i.e., chemicals of Unknown or Variable Composition, Complex Reaction Products and Biological Materials is an important aspect of the Dashboard. UVCBs can range from complex substances (e.g., tar or petroleum distillates) to a category of chemical substances whose members vary by chain lengths, substituent positions, etc., but they all share the property that they do not cleanly map to a single chemical structure. Given that many UVCBs are typically associated with industrial processes, effluents, etc., these substances are of particular interest to EPA’s TSCA program [147] and others [e.g. the NORMAN Network [148] ]. For instance, listed on the TSCA inventory is the substance “Light oil, coal, coke-oven” (CASRN: 65996-78-3). Ill-defined substances such as this can be registered to the DSSTox database and assigned a DTXSID (but not a DTXCID chemical identifier) and have associated information subsequently displayed on the Dashboard [149]. Clearly, a substance such as “Light oil, coal, coke-oven” is a complex mixture of hundreds if not thousands of chemicals. For UVCB chemicals, the ability to include chemical relationship mappings in the DSSTox database (referred to as predecessor and successor substances) allows the UVCB substance to be linked to substances that are represented by single chemical structures as Related Compounds. This is best exemplified by the substance “Alkylbenzenesulfonate, linear (CASRN: 42615-29-2)”, which lists 5 “Related Compound” structures on the Dashboard landing page [150]. Mass spectrometry studies have identified a number of these surfactant chemicals in Swiss wastewater [138]. Hence, manual curator mapping of these chemicals to the UVCB substance name in the DSSTox database allows for registered substances with defined structures to be displayed as related chemicals. As shown in Fig. 15, four of the five related chemicals are listed with “NOCAS” identifiers (below the structure), which are assigned within DSSTox when a CASRN was either not found or has not been assigned by CAS, which is sometimes the case with newly detected contaminants or transformation products. It should be noted that the surfactant itself, the class of linear alkylsulfonates, is contained within a list in the Dashboard: “Surfactant List Screened in Swiss Wastewater (2014) [151].

The value of these relationship mappings to MS work and NTA studies has been highlighted in a recent publication [145]. Ongoing support for UVCB chemicals is a particularly important area of focus for future work, as described below, in order to more fully support TSCA, other EPA programs, and ultimately support the global challenge of identifying complex substances in the environment. In particular, rather than perform the manual mappings between a UVCB chemical and existing chemical structures in the DSSTox database, initial developments for enumeration of Markush structures [152] and auto-mapping within the database [153] are already in place.

## Discussion and conclusions

The U.S. Environmental Protection Agency’s (EPA) web-based CompTox Chemistry Dashboard provides access to *high*-*quality, structure*-*curated, open data* to meet the various needs of the environmental sciences and computational toxicology communities. It integrates diverse types of relevant domain data including physicochemical, environmental fate and transport, exposure, usage, in vivo toxicity, and in vitro bioassay data. Batch searching allows for direct chemical identifier (ID) mapping and downloading of multiple data streams in several different formats and facilitates access to available structure, property, toxicity, and bioassay data for collections of thousands of chemicals at a time.

The Dashboard, publicly launched in April 2016, has expanded considerably in content and user traffic over the past year. The growth curve for usage has seen a 10 × increase in daily and monthly usage over a 1-year period with ~  1200 unique users per day, and ~  27,000 users per month as of November 2017. It is continuously evolving with the growth of DSSTox into high-interest or data-rich domains of interest to EPA, such as chemicals on the Toxic Substances Control Act (TSCA) listing, while providing the user community with a flexible and dynamic web-based platform for integration, processing, visualization and delivery of data and resources. The Dashboard provides support for a broad array of research and regulatory programs across the worldwide community of toxicologists and environmental scientists.

As purposely emphasized in this paper, the Dashboard is made up of a collection of databases that are integrated and surfaced through a single web-based interface via a set of tabs and subtabs using a chemical centric approach to integrating the data. The Dashboard architecture has, from the initial planning stages, been implemented in a manner that allows for additional modules and data streams to be readily and efficiently incorporated. This allows the quick introduction of new modules online using the appropriate data streams and visualization approaches. These new modules are commonly tested in-house for a few weeks prior to release to the community as “beta-modules”. As of August 2017, the ToxValDB and ADME tabs on the Dashboard are still labeled as Beta, while user feedback is gathered in order to help optimize the display and data for the user base. Also presently undergoing internal beta testing inside the EPA, and slated for future release to the public, is an implementation of “Generalized Read-Across” (GenRA) previously described by Shah et al. [154].

The Dashboard provides a portal to access many different data streams. For users interested in one type of data to address a specific question, this infrastructure is invaluable. However, there are other use cases where an integration or summary view of all the data streams could be useful to quickly capture the amount of available data, or the hazards or exposures that might be pertinent for risk assessment. Another aspect that is undergoing internal testing and refinement is an Executive Summary tab for a retrieved chemical substance. This provides a “one page” snapshot of salient attributes of the substance of interest. It is presently structured to report Quantitative Risk Assessment values, i.e. reference doses or toxicity values available within ToxValDB. A graph depicting the array of available toxicity values and their confidence intervals, where known, is also shown to quickly highlight which value might be the most conservative or whether the reported values are aligned with each other. The next set of summaries capture what endpoint specific information exists—namely to address carcinogenicity, repro-developmental, chronic toxicity and acute toxicity endpoints. Other headings are specific to organ toxicity effects, endocrine system effects, ADME, fate and transport, exposure. Finally, a representation of the ToxCast and EDSP assays is shown to showcase which toxicity pathways might be of concern.

As should be evident from this paper, data quality and curation are of foremost concern in the delivery of a web-based resource to serve environmental scientists and other potential users of the Dashboard. A great deal of attention is paid to data quality and curation within the DSSTox project, which has limited, to some extent, the degree of coverage of our chemistry database to the universe of chemicals of possible interest. However, at this time, it is the availability of data to be utilized in the Linked Data [155] and Semantic Web [156] that limits the overall impact of the resources underpinning the Dashboard. As described earlier, much of the Dashboard data is made available via the downloads page, and so is readily available to third party resources to consume. The DTXSID identifier has recently been accepted as a Wikidata Property [157] and this should help in exposing the Dashboard data to the expanding world of Big Data that can support chemical toxicity research [158]. Towards this end, future work associated with the Dashboard and its underlying data includes exposing an associated SPARQL endpoint [159].

In conclusion, we believe that the Dashboard, in its current form, provides a useful web application tool for accessing a broad array of databases, models, tools and capabilities. Although the main focus of EPA’s research is to support the Agency’s mission to evaluate chemical safety and protect human health and the environment, many data streams and capabilities surfaced in the Dashboard will have broader applicability across the chemical and biomedical research community. Additionally, not only is the Dashboard undergoing continuous growth and improvement as new data streams and capabilities are incorporated, but the Dashboard project is successfully partnering with and influencing the direction of wide-ranging EPA research projects in a more coordinated fashion, for the ultimate benefit of all parties concerned.

## Additional files

**Additional file 1.** BCF: The Fish Bioconcentration Factor from OPERA (OPEn saR App) models.

**Additional file 2.** List of data Sources in the External Links database.

## Abbreviations

ACToR: Aggregated Computational Toxicology Resource
ADME: absorption, distribution, metabolism and excretion
AOP: adverse outcome pathway
BAF: bioaccumulation factor
BCF: bioconcentration factor
BMD: benchmark dose
CAS-RN: CAS Registry Number
CPCat: Chemical and Product categories database
CPDat: Chemical and Products database
CSS: Cascading Style Sheets
DSSTox: Distributed Structure Searchable Toxicity database
DTXCID: DSSTox chemical identifier
DTXRID: DSSTox record identifier
DTXSID: DSSTox substance identifier
ECHA: European Chemicals Agency
EDSP: Endocrine Disruption Screening Program
ENTACT: EPA Non-Targeted Analysis Collaborative Trial
EPA: United States Environmental Protection Agency
EPI Suite: Estimation Program Interface Suite
EU: European Union
ExpoCast: Exposure Forecaster
GenRA: Generalized Read-Across
HSDB: Hazardous Substances Data Bank
InChI: International Chemical Identifier
InVitroDB: In Vitro database
JRC: Joint Research Centre
LEL: low effect level
LLNA: local lymph node assays
LO(A)EL: lowest observed (adverse) effect level
MSDS: Material Safety Data Sheet
NCBI: National Center for Biotechnology Information
NCCT: National Center for Computational Toxicology
NEL: no effect level
NICEATM: NTP Interagency Center for the Evaluation of Alternative Toxicological Methods
NIH: National Institutes of Health
NLM: National Library of Medicine
NO(A)EL: no observed (adverse) effect level
NTA: non-targeted analysis
OECD: Organisation for Economic Co-operation and Development
OPERA: Open SAR Application
PMID: PubMed ID
POD: Point-of-Departure
QSAR: quantitative structure activity relationship
QSUR: quantitative structure use relationship
REACH: registration, evaluation, authorisation and restriction of chemicals
RSL: regional screening level
REST: representational state transfer
RfC: reference concentration
RfD: reference dose
SAR: structure activity relationship
SMILES: simplified molecular-input line-entry system
SRS: EPA Substance Registry Service
TEST: EPA Toxicity Estimation Software Tool
ToxCast: Toxicity Forecaster
Tox21: Toxicology in the 21st Century program
TOXNET: TOXicology Data NETwork
ToxRefDB: ToxValDB: Toxicity Value Database
TSCA: Toxic Substances Control Act
