# Supplementary material for: The CompTox Chemistry Dashboard: a community data resource for environmental chemistry
Source: J Cheminform. 2017 Nov 28;9:61. doi: 10.1186/s13321-017-0247-6 (PMC5705535; doi:10.1186/s13321-017-0247-6)
Supplement: Supplementary file 2 — Additional file 2. List of data Sources in the External Links database. [file 13321_2017_247_MOESM2_ESM.docx]

**Additional file 2: List of data Sources in the External Links database**

| **Third-party**  **repository** | **Integration Identifier** | | | | |
| --- | --- | --- | --- | --- | --- |
|  | **CASRN** | **Chemical Name** | **Repository Identifier** | **InChIKey** | **SMILES** |
| **GENERAL RESOURCES** | | | | | |
| Substance Registry Service | ✓ | - | - | - | - |
| Household Products DB | ✓ |  |  | - | - |
| PubChem | - | - | ✓ | - | - |
| ChemSpider | - | - | ✓ | - | - |
| CPCat | ✓ | - | - | - | - |
| Wikipedia | - | ✓ | - | - | - |
| MSDS Lookup | ✓ | - | - | - | - |
| ChEMBL | - | - | ✓ | - | - |
| Chemical Vendors | ✓ | - | - | - | - |
| OEHHA Database | - | ✓ | - | - | - |
| NIOSH Safety Cards | - | - | ✓ | - | - |
| ToxPlanet | ✓ | - | - | - | - |
| ACS Reagent Chemicals | - | - | ✓ | - | - |
| Wikidata | - | - | ✓ | - | - |
| ChemHat | ✓ | - | - | - | - |
| Wolfram Alpha | - | ✓ | - | - | - |
| ECHA Brief Profile | - | - | ✓ | - | - |
| ECHA Infocard | - | - | ✓ | - | - |
| ChemAgora | ✓ | - | - | - | - |
| Consumer Product DB | ✓ | - | - | - | - |
| ChEBI | - | - | - | ✓ | - |
| Sigma-Aldrich Chemicals | ✓ | - | - | - | - |
| NIST Chemistry Web | ✓ | - | - | - | - |
| **TOXICOLOGY** | | | | | |
| ACToR | ✓ | - | - | - | - |
| DrugPortal | ✓ | - | - | - | - |
| CCRIS | ✓ | - | - | - | - |
| ChemView | ✓ | - | - | - | - |
| CTD | - | - | ✓ | - | - |
| eChemPortal | - | - | ✓ | - | - |
| Gene-Tox | - | - | - | - | - |
| HSDB | ✓ | - | - | - | - |
| ToxCast Dashboard 2 | ✓ | - | - | - | - |
| LactMed | - | - | - | - | - |
| ITER Database | ✓ | - | - | - | - |
| ATSDR | - | - | ✓ | - | - |
| Superfund SCDM | ✓ | - | - | - | - |
| NIOSH IDLH Values | ✓ | - | - | - | - |
| ACToR PDF Report | ✓ | - | - | - | - |
| Toxics Release Inventory | ✓ | - | - | - | - |
| CREST | - | - | - | ✓ | - |
| **PUBLICATIONS** | | | | | |
| Toxline | ✓ | - | - | - | - |
| Env. Health Perspectives | ✓ | - | - | - | - |
| NIEHS | ✓ | - | - | - | - |
| National Tox. Program | ✓ | - | - | - | - |
| Google Books | ✓ | ✓ | - | - | - |
| Google Scholar | ✓ | ✓ | - | - | - |
| Google Patents | ✓ | ✓ | - | - | - |
| PubMed | ✓ | - | - | - | - |
| IRIS Assessments | - | - | ✓ | - | - |
| NIOSH Pocket Guide | - | - | ✓ | - | - |
| RSC Publications | ✓ | - | - | - | - |
| BioCaddie DataMed | - | ✓ | - | - | - |
| Springer Materials | - | - | - | ✓ | - |
| Federal Register | - | ✓ | - | - | - |
| Regulations.gov | - | ✓ | - | - | - |
| Bielefeld Search Engine | - | ✓ | - | - | - |
| CORE Literature Search | - | ✓ | - | - | - |
| **ANALYTICAL** | | | | | |
| Nat. Enviro. Methods Index | ✓ | - | - | - | - |
| RSC Analytical Abstracts | ✓ | - | - | - | - |
| Tox21 Analytical Data | ✓ | - | - | - | - |
| MassBank North America | - | - | - | ✓ | - |
| mzCloud | - | - | ✓ | - | - |
| NIST IR Spectrum | ✓ | - | - | - | - |
| NIST MS Spectrum | ✓ | - | - | - | - |
| **PREDICTION** | | | | | |
| 2D HSQC/HMBC Pred. | - | - | - | - | ✓ |
| Carbon-13 NMR Pred. | - | - | - | - | ✓ |
| Proton NMR Pred. | - | - | - | - | ✓ |
| ChemRTP Predictor | - | - | - | ✓ | - |
